# L-2-Hydroxyglutarate sensitizes ferroptosis through ATF3/CHAC1-mediated glutathione degradation in hepatocellular carcinoma

**DOI:** 10.1038/s41419-026-09052-x

**Published:** 2026-07-08

**Authors:** Caixia Xi, Junfeng Pang, Mohammad Oliaeimotlagh, Rui Mao, Wenbo Zhi, Kimya Jones, Tianxiang Hu, Huidong Shi, Ande Satyanarayana, Anatolij Horuzsko, Chunhong Yan, Nahid F. Mivechi, Xingguo Zhu

**Affiliations:** 1 Georgia Cancer Center, Augusta University, Augusta, GA USA; 2 Department of Pediatrics, Augusta University, Augusta, GA USA; 3 Institute for Immunology and Immune Health, Perelman School of Medicine, University of Pennsylvania, Philadelphia, PA USA; 4 Center for Biotechnology and Genomic Medicine, Augusta University, Augusta, GA USA; 5 Department of Pathology, Medical College of Georgia, Augusta University, Augusta, GA USA; 6 Department of Radiation Oncology, Augusta University, Augusta, GA USA

**Keywords:** Cancer metabolism, Apoptosis

## Abstract

Ferroptosis is an iron-dependent form of regulated cell death driven by lipid peroxidation and glutathione (GSH) depletion and represents a therapeutic vulnerability in hepatocellular carcinoma (HCC). While canonical ferroptosis regulation centers on cystine uptake and GPX4-mediated GSH utilization, the endogenous metabolic pathways governing ferroptosis sensitivity in liver tumors remain incompletely understood. Here, using a metabolic-scale CRISPR activation screen integrated with transcriptomic and metabolomic analyses, we identify L-2-hydroxyglutarate dehydrogenase (L2HGDH) as a potent antagonist of ferroptosis in HCC. We demonstrate that L2HGDH is frequently suppressed in liver tumors, leading to pathological accumulation of its substrate L-2-hydroxyglutarate (L2HG). Elevated L2HG sensitizes HCC cells to ferroptosis both in vitro and in vivo. Mechanistically, L2HG acts as a metabolic-epigenetic regulator that inhibits 2-oxoglutarate-dependent dioxygenases, induces histone hypermethylation, and remodels chromatin accessibility to activate an ATF3-dependent transcriptional program. This program induces the glutathione-degrading enzyme CHAC1, thereby accelerating GSH degradation to 5-oxoproline and disrupting redox homeostasis. Notably, L2HG-induced ferroptosis occurs independently of impaired cystine uptake, transsulfuration pathway activity, or increased GPX4-mediated GSH utilization, revealing a non-canonical ferroptosis mechanism driven by enhanced GSH catabolism. Consistent with these findings, genetic targeting of L2HGDH suppresses tumor growth, elevates L2HG levels, enhances GSH degradation, and promotes ferroptosis in HCC xenograft models. Collectively, our study identifies the L2HGDH-L2HG axis as a previously unrecognized metabolic checkpoint controlling ferroptosis sensitivity in liver cancer and uncovers glutathione degradation as a therapeutically exploitable vulnerability for ferroptosis-based treatment strategies in HCC.

## Highlights

- A metabolic-scale CRISPR Activation (CRISPRa) screen identifies L2HGDH as a ferroptosis antagonist.
- L2HG sensitizes HCC cells to ferroptosis through ATF3/CHAC1-mediated GSH degradation.
- L2HG promotes chromatin accessibility through histone hypermethylation for gene regulation.
- *L2HGDH* silencing inhibited tumor xenograft growth in vivo.

## Graphical abstract

**Figure:**
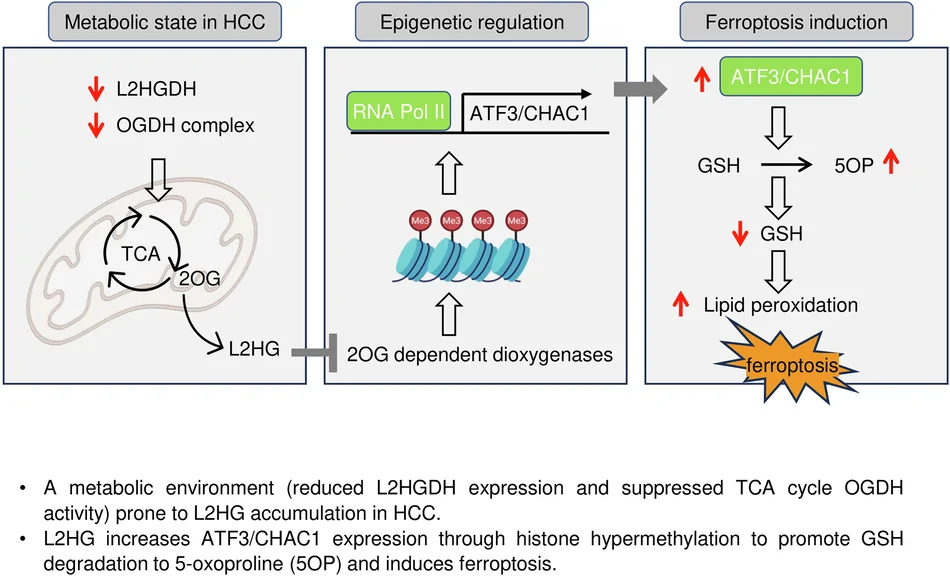
A metabolic environment (reduced L2HGDH expression and suppressed TCA cycle OGDH activity) prone to L2HG accumulation in HCC. L2HG increases ATF3/CHAC1 expression through histone hypermethylation to promote GSH degradation to 5-oxoproline (5OP) and induces ferroptosis.

## Introduction

Ferroptosis is a non-apoptotic form of regulated cell death, characterized by excessive iron accumulation and lipid peroxidation. Over the last decade, ferroptosis has been extensively investigated across diverse biological contexts, with therapeutic strategies aiming either to inhibit ferroptosis to mitigate tissue injury or to induce ferroptosis to suppress malignant cell proliferation. With biochemical feature of iron accumulation, lipid peroxidation, glutathione shortage, ferroptosis is regulated by factors mediating 1) cysteine/cystine availability (such as SLC7A11, SLC3A2, ASCT1/2, CDO1, etc.), 2) glutathione (GSH) synthesis (GCLC, GSS, etc.), 3) GSH homeostasis and peroxidation (CHAC1, GGT, GPX4, GPX4 expression/activity regulation: mTOR, DHODH), 4) lipid metabolism (ACSL4) and 5) iron metabolism (CP, HEPH, FTH, etc.) (reviewed [1–5]). In addition, the de novo cysteine synthesis through the folate and methionine cycles, such as GCH1, [6] SHMT/SHMT2, [7, 8] GNMT [9] AHCY [9, 10], critically contributes to ferroptosis regulation, particularly in the liver and brain, which exhibit high transsulfuration activity.

Ferroptosis plays a multifaceted role in liver disease progression, transitioning from non-alcoholic fatty liver disease (NAFLD), non-alcoholic steatohepatitis (NASH), cirrhosis, and eventually hepatocellular carcinoma (HCC). Notably, ferroptosis has been implicated in promoting NAFLD and NASH while potentially suppressing cirrhosis and HCC. [11, 12] This context-dependent behavior has prompted both pro- and anti-ferroptosis strategies as therapeutic approaches for liver diseases [13, 14]. These observations highlight the need to further elucidate ferroptosis regulatory mechanisms in HCC to improve ferroptosis-based therapeutic strategies.

In this study, we sought to identify ferroptosis-regulatory metabolites and their underlying mechanisms using a metabolic library screen integrated with transcriptomic and metabolic network analyses. We identified L-2-hydroxyglutarate (L2HG) and its metabolic enzyme L-2-hydroxyglutarate dehydrogenase (L2HGDH) as key regulators of ferroptosis. L2HG accumulates abnormally in HCC due to L2HGDH suppression and dysregulated tricarboxylic acid (TCA) cycle activity. Elevated L2HG induces histone hypermethylation and alters chromatin accessibility at ferroptosis-related genes, thereby activating the ATF3/CHAC1 axis to disrupt GSH homeostasis. Importantly, we demonstrate that L2HG promotes ferroptosis primarily by accelerating GSH degradation to 5-oxoproline (5OP, pyroglutamic acid), rather than by increasing GSH utilization, impairing cystine uptake, or suppressing transsulfuration pathway activity. This effect is mediated through ATF3-dependent induction of CHAC1 following L2HG-driven epigenetic reprogramming. Collectively, our findings establish the L2HGDH/L2HG axis as a previously unrecognized regulator of ferroptosis that controls cellular proliferation and viability in liver tumor cells. These insights reveal a metabolic vulnerability that may be therapeutically exploited for ferroptosis-targeted strategies in HCC.

## Materials and methods

### Human specimens and ethics

Liver and tumor tissues were obtained from patients with HCC following written informed consent. Samples were provided by the UC Davis Health Biorepository Shared Resource under a protocol approved by the Institutional Review Board of Augusta University. Patient demographics, including age and sex, are summarized in Supplementary Table 1. All samples were snap-frozen and stored at -80 °C. The authors had no access to identifiable patient information.

### Cell lines and culture

The HCC cell lines HepG2, HepG2/C3A, PLC/PRF/5, Hep3B, HuH7, SNU423, SNU398, SNU449, SNU475, and SK-HEP1 were obtained from the American Type Culture Collection (Manassas, VA, USA) or generously provided by Dr. M. Celeste Simon (University of Pennsylvania). All cell lines were tested quarterly for mycoplasma contamination using PCR-based analysis using primers (Supplemental Table 2). For cell lines under puromycin or blasticidin selection, antibiotics were removed 48 h prior to experiments.

All the HCC cells were cultured in Human Plasma-Like Medium (HPLM, ThermoFisher) supplemented with 10% dialyzed FBS (HyClone). For amino-acid isotopic tracing, cells were cultured in customized HPLM prepared according to the published formulation [15] (Supplemental Table 3) containing all 20 amino acids except cystine or serine, as indicated.

### Tumor xenograft experiments

Immunodeficient NOD.Cg-*Prkdc*^*scid*^ *Il2rg*^*tm1Wjl*^/SzJ (NSG) mice (the Jackson laboratory), aged 2.5–3 months, were used for liver tumor xenograft studies. Briefly, 2 × 10^6^ Hep3B cells were subcutaneously injected into the right flank of each mouse. Tumor volume was measured every other day and calculated using the formula: v = (length x width^2^)/2. All animal procedures were conducted in accordance with federal ethical guidelines and were approved by the Institutional Animal Care and Use Committee of Augusta University.

### Chemicals

Erastin, Ferrostatin-1 and deferoxamine mesylate from ApexBio (Houston, TX); Imidazole ketone erastin (IKE), Z­VAD(OMe)­FMK, (±)­Necrostatin­2, Bafilomycin A1, (1S,3R)-RSL3 from Cayman Chemicals (Ann Arbor, MI), TFMB-L2HG from Sigma (St. Louis, MO) were used at the indicated concentrations.

### Metabolism-focused CRISPRa screen

A metabolism-focused CRISPR activation (CRISPRa) sgRNA library targeting 2989 metabolic genes with a total of 32,509 sgRNAs [16] was used. Lentiviral particles were generated by co-transfecting the library plasmid pool with pMD2.G and psPAX2 packaging plasmids into HEK293FT cells.

HuH7 cells were transfected with SP-dCas9-VPR plasmid and selected with G418 (Corning) for stable transfectants and then transduced with the lentiviral CRISPRa sgRNA library. Two days post-transduction, 1 × 10^7^ cells were harvested for genomic DNA (gDNA) extraction, while the remaining cells were cultured in HPLM medium containing IKE. After 14 days, an additional 1 × 10^7^ cells were collected for gDNA extraction (final population) for sgRNA sequencing analysis. Sequencing reads were aligned to the reference sgRNA library, and sgRNA abundance was quantified. Results were expressed as percentage total sgRNA reads for each gene.

### RNA sequencing

Total RNA was extracted using the PureLink RNA Mini Kit (Invitrogen). cDNA libraries were prepared using the NEBNext Ultra RNA Library Prep Kit (New England Biolabs) and sequenced on an Illumina NextSeq 500 platform. FASTQ files were processed using the RNA Express module (Illumina). Reads were aligned to the human genome (GRCh38/hg38) using STAR. Normalization and differential gene expression analyses were performed in R (v4.0.3) using the edgeR package.

### ATAC-seq

ATAC-seq library preparation was performed using the ATAC-seq kit (Active Motif, Cat# 53150) following the manufacturer’s instructions. Hep3B cells were cultured in HPLM and treated with or without 2 mM TFMB-L2HG. For each condition, 50,000 cells were harvested, washed with ice-cold PBS, lysed, and nuclei were collected by centrifugation at 500 × *g* for 10 min at 4 °C. Tagmentation, library amplification, and purification were performed according to the kit protocol. Library quality was assessed using the Agilent High Sensitivity DNA Kit. Libraries were sequenced on an Illumina NextSeq 500 platform. Reads were aligned to the human genome (GRCh37/hg19) using Bowtie2, retaining uniquely mapped reads. Chromatin accessibility peaks were identified using HOMER and Genrich v0.5, and data were visualized using UCSC Genome Browser and IGV.

### Genomics data of HCC cell lines and human tumors

RNA-seq data for HCC cell lines and human liver tumors were retrieved from the Cancer Cell Line Encyclopedia (CCLE) dataset and The Cancer Genome Atlas (TCGA) dataset, including 50 normal liver and 366 HCC samples, expressed as log_2_(TPM + 1). Gene Set Enrichment Analysis (GSEA) was used for pathway enrichment. Pearson correlation coefficients and associated *p* values were computed using MATLAB.

### Human liver tumor protein expression and survival analysis

Protein expression data (normalized relative protein expression, nRPX) were obtained from the Human Protein Atlas (HPA) dataset [17], which includes tumor and matched normal liver tissues from 165 patients with available clinical outcomes. Kaplan-Meier survival analyses were performed using GraphPad Prism (v9.0.0).

### Plasmids constructs

All plasmids generated in this study were verified by Sanger sequencing and deposited in Addgene (Watertown, MA), including sgControl, sg*L2HGDH*, sh*GPX4*, sh*OGDH*, sh*DLD*, sh*DLST*, sh*CHAC1*, sh*ATF3*, sh*KDM6B*, L2HGDH^OPT^, OGDH^OPT^, ATF3^OE^ and CHAC1 promoter constructs. See Supplemental Table 4 for plasmid details.

### Cell viability and proliferation assays

Cell viability was measured using the Cell Counting Kit-8 (CCK-8; ApexBio) with absorbance read at 450 nm. For proliferation assays, GFP-expressing HCC cells were generated using pGreenFire1 (System Biosciences). Cells were seeded at 5000-8000 cells per well and treated with TFMB-L2HG (0-2 mM). Proliferation was monitored using the Incucyte Live-Cell Analysis System (Sartorius) and quantified by GFP fluorescence and confluency.

Kinetic ferroptotic cell death signals were evaluated as reported [18]. Briefly, Sytox™ Green (S7020, ThermoFisher Scientific) was added to the wells in a final concentration of 10 nM. Cell death was recorded over time with the IncuCyte platform at 37 °C and 5% CO2 using a 20x objective. Phase contrast and green fluorescence (Ex/Em: 504 nm/523 nm, 200 ms exposure) images were taken at 4 h of interval. To reduce intercellular connection, HCC cell lines were seeded at 1000–2000 cells (depending on the cell line) per well in 96-well plates. To calculate the percentage of Sytox Green-positive cells, the Sytox Green-positive area was divided by the total cell area measured from phase-contrast images at each time point.

### Metabolite profiling and isotope tracing

Intracellular metabolites were extracted from 3 × 10^6^ cells or 40 mg tumor tissue using ice-cold 80% methanol and analyzed by LC-MS/MS using a Q Exactive Plus mass spectrometer coupled to an Ultimate 3000 UHPLC system as reported [19]. Metabolite identification was performed using MassHunter Quant with reference libraries. Data analysis was conducted using MetaboAnalyst 6.0.

For isotope tracing, Hep3B cells were cultured in HPLM medium lacking either cystine or serine for 2 h and then incubated with 100 μM 3,3’-^13^C_2_ cystine or 1-^13^C serine (Cambridge Isotope Laboratory). Following the tracing period, metabolites were extracted and metabolite quantification was conducted [20]. Isotopomer distributions were corrected for natural isotope abundance and normalized to protein content.

### Quantification of 2HG enantiomers (D2HG and L2HG)

Quantification of D2HG and L2HG was performed by LC-MS/MS following derivatization with N-(p-Toluenesulfonyl)-L-phenylalanyl chloride (TSPC) (ThermoFisher), as reported [20]. Final 2HG values were normalized to cell numbers or tissue mass.

### Measurement of 2-oxoglutarate (2OG)

Intracellular 2OG levels were measured using the Alpha-Ketoglutarate Assay Kit (Abcam), following the manufacturer’s protocol. Absorbance was measured at 340 nm with concentrations calculated by linear regression using standard curves.

### Measurement of 5-oxoproline (5OP)

Approximately 3 × 10^6^ cells were extracted in ice-cold 80% methanol containing 1 μM ^13^C_3_ lactate (internal standard), homogenized, centrifuged, and vacuum dried. The extracts were reconstituted in 20% methanol and analyzed by LC-MS/MS with Multiple Reaction Monitoring (MRM) (LC-MRM MS) using a Shimadzu Nexera UHPLC coupled to a TSQ Quantiva triple-quadrupole mass spectrometry with positive ESI. Peak areas were quantified using Skyline and normalized to the internal standard.

### Lipid peroxidation assay using Bodipy-C11

HCC cells under different treatments were incubated with 2 μM Bodipy-C11 (Invitrogen) for 30 minutes at 37 °C. Cells were then trypsinized and washed once with PBS. Cellular fluorescence was then measured by flow cytometry analysis.

### Lipid peroxidation malondialdehyde (MDA) assay

Lipid peroxidation levels were measured using MDA assay kit (Sigma) following the manufacturer’s instructions and normalized to total protein content.

### Glutathione assay

Reduced and oxidized glutathione levels were measured using a GSH/GSSG assay kit (BioAssay Systems). Absorbance was measured at 412 nm and results were normalized to total cellular protein content or tissue mass.

### Intracellular iron FerroOrange measurement

Intracellular Fe²^+^ was measured using FerroOrange (Dojindo). Cells were incubated with 1 µM probe for 20 min at 37 °C and analyzed by fluorescence microscopy or flow cytometry.

### Cystine uptake assay

Cystine uptake was measured using the BioTracker™ Cystine-FITC Live Cell Dye (Sigma) analyzed by flow cytometry to quantify intracellular Cystine-FITC levels.

### Histone H3 modification analysis by ELISA

EpiQuik Histone H3 Modification Multiplex Assay Kit (Epigentek) was used. Equal amounts of histone extracts were added for each sample and H3 modifications were calculated according to manufacturer’s instructions, after normalized to protein content. The experiment is performed in monoplicate.

### mRNA expression analysis

Total RNA was extracted using the RNeasy Mini Kit (Invitrogen). cDNA was synthesized using cDNA Synthesis Kit (Bio-Rad). Relative mRNA expression levels were calculated using the ΔΔCt method and normalized to internal β-actin levels. Primers used for PCR are listed in Supplementary Table 2.

### Immunoblotting

Approximately 15 μg whole cell lysate or 2 μg histone extract was resolved on 10% SDS-PAGE gels and transferred to nitrocellulose membranes. Membranes were blocked in 5% non-fat milk and incubated with appropriate primary and HRP-conjugated secondary antibodies (Supplementary Table 5). Signal was detected by chemiluminescence, and image quantifications were conducted with ImageJ.

### Immunohistochemistry

Five micrometer sections from FFPE tissues were stained with H&E, or primary antibodies followed by incubation with ImmPRESS Excel Amplified Polymer Staining Kit and counterstained with Hematoxylin QS (Vector Laboratories).

To determine the ferroptosis by FerroOrange staining in the xenografts, snap-frozen tissues at 5 µm sections were stained with FerroOrange for 30 min. After PBS wash twice, FerroOrange staining was imaged under fluorescence with DAPI nuclei staining.

### OGDH activity assay

Oxoglutarate dehydrogenase (OGDH) activity was measured using the OGDH Activity Assay Kit (Sigma). Briefly, tissues were homogenized in ice-cold OGDH assay buffer and the homogenates were centrifuged. The supernatants were then collected and incubated with the assay reaction mix, and absorbance at 450 nm was measured in kinetic mode.

### Chromatin Immunoprecipitation (ChIP) assays

ChIP assays were performed using the ChIP Assay Kit (Sigma) as reported [19] with antibodies against ATF3, TBP, RNA Polymerase II (Pol II), H3K4Me3, H3K9Me3, and H3K27Me3. Normal rabbit IgG was used as a negative control. Immunoprecipitated DNA was purified and analyzed by quantitative PCR using gene-specific primers (Supplementary Table 2).

### CHAC1 promoter activity assay

Human CHAC1 wildtype promoter (-592bp ~ -10bp upstream the translation start codon) was synthesized by Twist Biosciences and cloned into pEGFP-N1 (Clontech, PT3027-5) in which the CMV promoter was removed. The ATF/CRE mutant was introduced by DNA synthesis for the CHAC1 mutant promoter. Both CHAC1 wildtype and mutant promoter driven GFP expression plasmids were transiently co-transfected with pDsRed1-N1 to Hep3B and determined promoter activities under L2HG treatment, in sh*L2HGDH*,sh*ATF3* or ATF3 overexpression cells and followed by flow cytometry analyses. GFP levels were normalized to transfection control DsRed (RFP) levels.

### Statistical analyses

Data are presented as mean ± SD from at least three independent biological replicates unless otherwise indicated. Statistical significance was determined using unpaired two-tailed Student’s *t*-tests. Analyses were performed using GraphPad Prism 9 or MetaboAnalyst, with *p* < 0.05 considered statistically significant.

## Results

### A metabolic scale CRISPR activation screen identifies L2HGDH as a ferroptosis antagonist

**Fig. 1:**
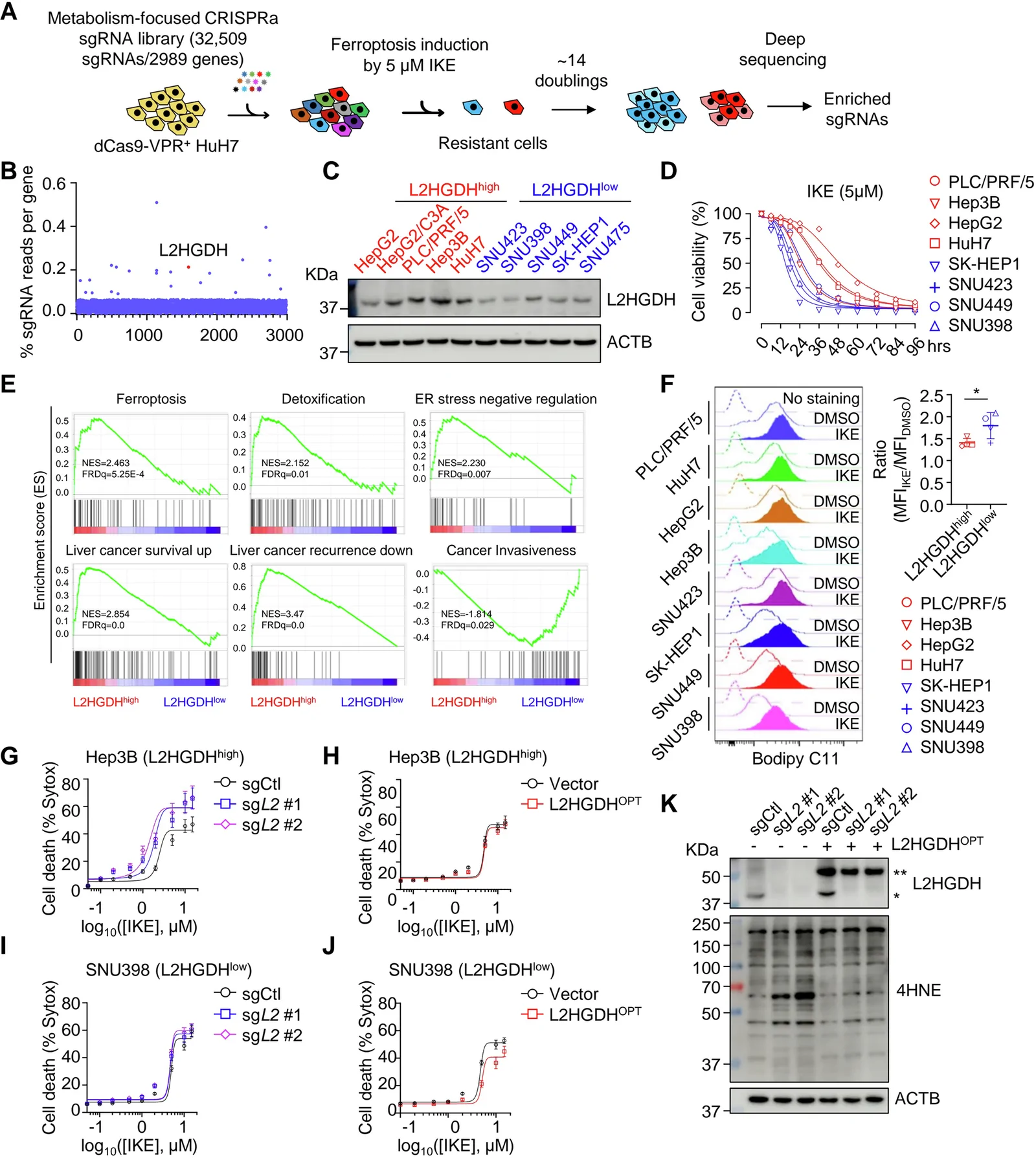
Identification of L2HGDH as a ferroptosis antagonist. **A** Schematic of a metabolism-focused CRISPRa screen performed in dCas9-VPR^+^ HuH7 cells under ferroptosis induction with 5 μM IKE treatment. **B** L2HGDH was identified as a ferroptosis antagonist. Data were plotted as genes in alphabetical order versus the percentage of sgRNAs reads per gene. The percentage of total sgRNA reads were calculated by summing up all sgRNA reads. **C** Immunoblotting analysis of L2HGDH expression across 10 human HCC cell lines, with high expression designated as L2HGDH^high^ and low expression as L2HGDH^low^. **D** Relative viability of L2HGDH^high^ and L2HGDH^low^ HCC cell lines following treatment with 5 µM of IKE for the indicated times. **E** Transcription pathway analysis comparing L2HGDH^high^ (HepG2, PLC/PRF/5, HuH7, Hep3B) and L2HGDH^low^ (SNU423, SNU449, SNU475, and SK-HEP1) HCC cell lines using RNA-seq data retrieved from the Cancer Cell Line Encyclopedia (CCLE) dataset. **F** Lipid peroxidation assessed by BODIPY-C11 staining in L2HGDH^high^ and L2HGDH^low^ cell lines under IKE (5 μM) or DMSO. Ratios of mean fluorescence intensity (MFI) (IKE/DMSO) were shown. **G**–**J** Incucyte Sytox green staining determined the cell death induced by different concentrations of IKE under *L2HGDH* silencing (sg*L2HGDH*, sg*L2*) or ectopic L2HGDH^OPT^ expression in L2HGDH^high^ Hep3B and L2HGDH^low^ SNU398 cells. Two independent constructs sg*L2* were tested. **K** Immunoblotting analysis of 4HNE protein adducts following sg*L2HGDH* silencing or L2HGDH^OPT^ expression. The data in (**D**, **F–J**) are presented as mean ± SD of three independent biological experiments. *p < 0.05, by unpaired Student’s *t* test.

To identify anti-ferroptosis genes in HCC, we performed a CRISPR activation (CRISPRa) screen. A HCC HuH7 cell line stably expressing nuclease-deactivated Cas9 fused to transcriptional activation domains (VP64, p65, and Rta; dCas9-VPR) [21], was generated and transduced with a metabolic CRISPRa sgRNA library targeting the promoters of 2,989 human metabolic genes [16]. Library-infected cells were cultured in human plasma-like medium (HPLM), which closely recapitulates the metabolite composition of human plasma [15], and treated with near-lethal doses of imidazole ketone erastin (IKE, 5 μM) to induce ferroptosis (Fig. 1A). After 14 days, surviving cells were collected and sgRNAs were sequenced to identify genes conferring resistance to ferroptotic stress. Notably, sgRNAs targeting the promoter region of *L2HGDH* gene were significantly enriched in the surviving population (Fig. 1B, Supplemental Table 6), suggesting that L2HGDH activation confers survival advantages under ferroptosis.

To validate these findings, we stratified a panel of HCC cell lines based on L2HGDH expression. HepG2, HepG2/C3A, PLC/PRF/5, HuH7, and Hep3B cells exhibited high L2HGDH expression (L2HGDH^high^), whereas SNU423, SNU398, SNU449, SNU475, and SK-HEP1 cells showed low expression (L2HGDH^low^) (Fig. 1C). Consistently, L2HGDH^high^ cells were significantly less sensitive to ferroptosis inducers (IKE and RSL3), while L2HGDH^low^ cells were markedly more sensitive (Fig. 1D, Supplementary Fig. 1A).

Subsequent transcriptomic analysis using RNA-seq data from the Cancer Cell Line Encyclopedia (CCLE) revealed distinct expression patterns between L2HGDH^high^ and L2HGDH^low^ cells in pathways related to ferroptosis, oxidative stress detoxification, endoplasmic reticulum stress, tumor survival, recurrence, and invasiveness (Fig. 1E). Moreover, L2HGDH^low^ cells exhibited higher lipid peroxidation, as indicated by increased IKE/DMSO ratios of Bodipy-C11 mean fluorescence intensity compared to L2HGDH^high^ cells (Fig. 1F), suggesting ferroptosis-related roles of L2HGDH.

To directly assess the role of L2HGDH in ferroptosis regulation, we knocked out L2HGDH using CRISPR/Cas9 (sg*L2HGDH*) in L2HGDH^high^ Hep3B cells (Supplementary Fig. 1B). Notably, L2HGDH loss significantly suppressed cell proliferation (Supplementary Fig. 1D–E), and increased sensitivity to IKE or RSL3-induced ferroptosis (Fig. 1G; Supplementary Fig. 1F, Supplementary Fig.1H). These effects were fully rescued by ectopic expression of a codon-optimized, sgRNA-resistant L2HGDH construct (L2HGDH^OPT^, with wobble mutations introduced) (Fig. 1H; Supplementary Fig. 1C–G, Supplementary Fig. 1I).

In contrast, sg*L2HGDH* had no detectable effect on ferroptosis sensitivity in L2HGDH^low^ SNU398 cells (Fig. 1I; Supplementary Fig. 1F, J), whereas L2HGDH^OPT^ expression conferred significant protection against ferroptosis (Fig. 1J; Supplementary Fig. 1K). Consistent with these findings, BODIPY-C11 staining demonstrated increased lipid peroxidation following L2HGDH loss in Hep3B cells but not in SNU398 cells, while L2HGDH^OPT^ reduced lipid peroxidation in both cell lines (Supplementary Fig. 1L–M). Similarly, levels of 4-hydroxynonenal (4-HNE) protein adducts were increased by sg*L2HGDH* and decreased by L2HGDH^OPT^ expression (Fig. 1K). Together, these results demonstrate that high L2HGDH expression confers resistance to ferroptosis, whereas low L2HGDH expression increases ferroptosis sensitivity, establishing L2HGDH as a robust ferroptosis antagonist whose expression inversely correlates with ferroptosis susceptibility in HCC cells.

### Vulnerable metabolic environment underlies L2HG accumulation in HCC

**Fig. 2:**
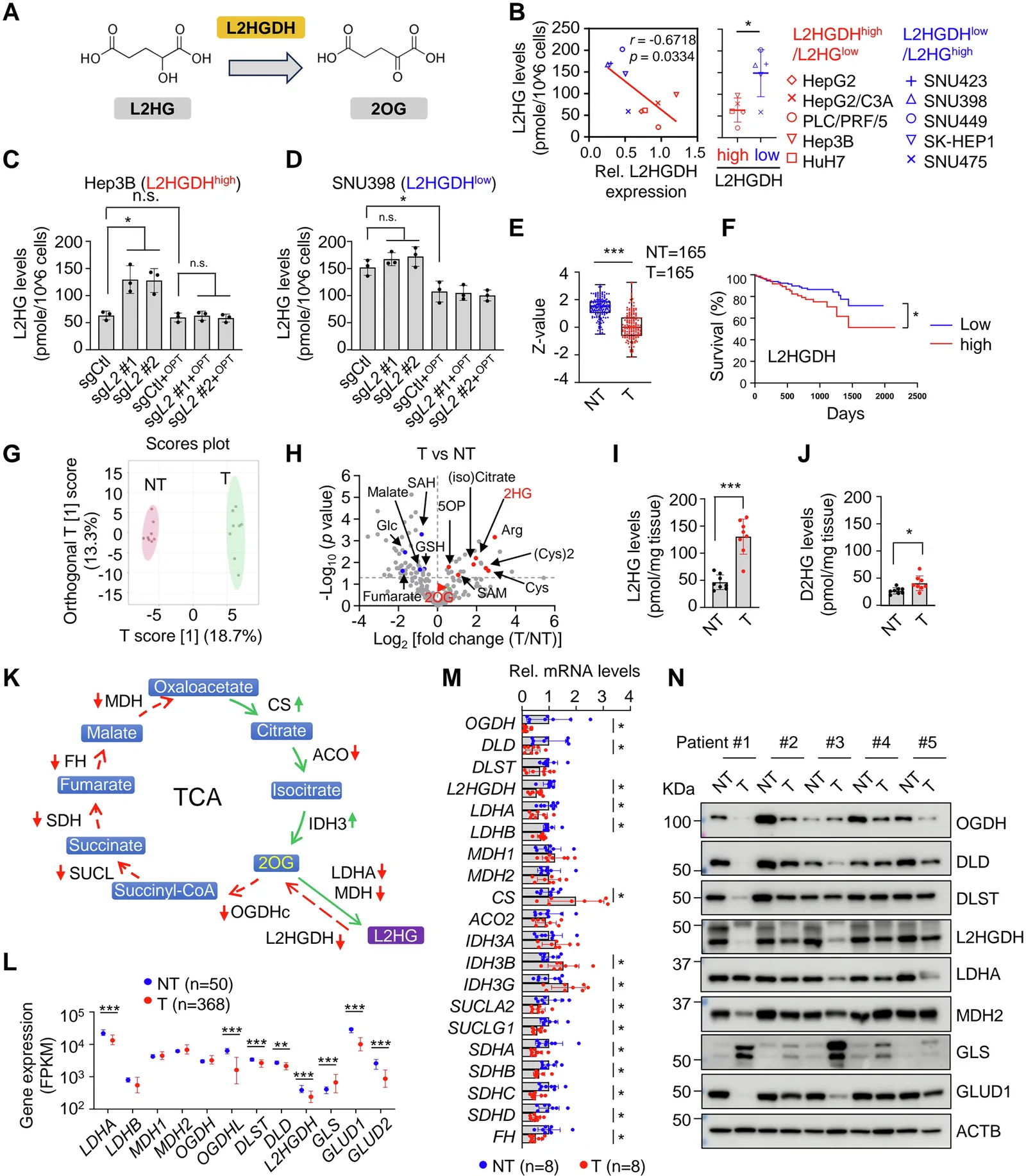
HCC exhibits a metabolic regulatory network that promotes L2HG accumulation. **A** Schematic illustration of L2HGDH catalyzing the oxidation of L2HG to 2-oxoglutarate (2OG). **B** Negative correlation between L2HGDH expression and intracellular L2HG levels across a panel of 10 HCC cell lines. L2HGDH^high^ HCC cells exhibit low L2HG levels, whereas L2HGDH^low^ cells accumulate L2HG. **C**, **D** Effect of L2HGDH silencing (sg*L2HGDH*) or ectopic L2HGDH expression (L2HGDH^OPT^) on L2HG levels in Hep3B (L2HGDH^high^) and SNU398 (L2HGDH^low^) cells. **E** L2HGDH protein expression in human liver tumor (T) and matched non-tumor (NT) tissues. Data was retrieved from the Clinical Proteomic Tumor Analysis Consortium (CPTAC) dataset (*n* = 165 per group). **F** Kapan–Meier overall survival analysis of HCC patient stratified by L2HGDH expression using data retrieved from the Human Protein Atlas. **G**, **H** LC-MS/MS analysis on metabolite profiles of human HCC tumors (T) and adjacent non-tumor (NT) tissues, shown as principal component analysis (PCA) (**G**) and Volcano plot analysis (**H**). Fold change (T/NT; *x*-axis) and statistical significance (*t*-tests, *y*-axis) were calculated using MetaboAnalyst 6.0 (*n* = 8 per group; *p* < 0.05). Chiral derivatization and LC-MS/MS quantification of L2HG (**I**) and D2HG (**J**) enantiomers in human liver T vs NT tissues (*n* = 8). **K** Model depicting metabolic rewiring leading to L2HG accumulation in HCC. Green arrows indicate enzymes upregulated in tumors, and red arrows indicate downregulated enzymes (expression data from the Human Protein Atlas; Supplementary Fig. 2C). Suppressed activity of the oxoglutarate dehydrogenase complex (OGDHc) increases 2OG availability, while reduced L2HGDH expression limits L2HG oxidation, together promoting L2HG accumulation. **L** mRNA expression levels (FPKM) of genes encoding enzymes involved in L2HG generation in human liver T vs NT tissues from the TCGA-LIHC dataset (T, *n* = 368; NT, *n* = 50). **M**, **N** RT-PCR and immunoblotting analyses of enzymes involved in the L2HG generation in paired human HCC T vs NT samples. The data is presented as mean ± SD (*n* = 5–8). Statistical significance was determined using unpaired two-tailed Student’s t tests or log-rank tests (survival analysis). Abbreviations: 2HG, 2-hydroxyglutarate; Glc, glucose; Cys, cysteine; (Cys)_2_, cystine; SAM, S-adenosylmethionine; SAH, S-adenosylhomocysteine; 5OP, 5-oxoproline; 2OG, 2-oxoglutarate; PCA, principal component analysis.

Functionally, L2HGDH catalyzes the oxidation of L-2-hydroxyglutarate (L2HG) to 2-oxoglutarate (2OG) (Fig. 2A). In agreement with this function, we observed negative correlation between L2HGDH expression and L2HG levels across L2HGDH^high^ and L2HGDH^low^ HCC cell lines (Fig. 2B). In L2HGDH^high^ Hep3B cells, sg*L2HGDH* significantly increased L2HG levels, an effect reversed by L2HGDH^OPT^ expression (Fig. 2C). In contrast, this regulation was not evident in L2HGDH^low^ SNU398 cells, although L2HGDH^OPT^ expression alone reduced L2HG levels (Fig. 2D), indicating L2HGDH expression-dependent control of L2HG abundance.

We next assessed whether there is clinical relevance for the expression of L2HGDH and L2HG levels. By utilizing the Clinical Proteomic Tumor Analysis Consortium (CPTAC) data, we observed significant downregulation of L2HGDH in liver tumors compared to normal tissues (Fig. 2E), with high L2HGDH expression correlating with poorer patient survival (Fig. 2F).

Subsequent LC-MS/MS analysis on a cohort of patient liver tumor samples further revealed dysregulated arginine metabolism, consistent with prior studies [22–26] alongside alterations in methionine cycle, transsulfuration, cystine uptake, and GSH metabolism between tumors and non-tumors (Fig. 2G, H, Supplementary Fig. 2A–B, Supplemental Table 7). Notably, total 2HG levels were markedly elevated in tumors (Fig. 2H, Supplementary Fig. 2A), implicating a potential tumor-relevant role. Since 2HG exists as two enantiomers: D-2-hydroxyglutarate (D2HG) and L2HG, which are indistinguishable by conventional LC-MS/MS, we employed a chiral derivatization by LC-MS/MS to differentiate them [20]. This analysis revealed elevation of both D-2HG and L2HG in tumors, with L2HG being the predominant one (Fig. 2I, J).

L2HG itself is produced by the promiscuous activity of LDHA and MDH1/2 under hypoxic or acidic conditions and is converted back to 2OG by L2HGDH [27–29]. In addition, structurally like 2OG, 2HG is generated by reducing the ketone group of 2OG to a hydroxyl group and the availability of 2OG influences L2HG levels (Fig. 2K). Therefore, we next explored whether these factors are affected to regulate L2HG levels. In tumors, 2OG levels were marginally increased (*p* > 0.05) (Fig. 2H).

Further analysis of TCGA-LIHC, Human Protein Atlas, and cohorts of patient samples revealed significant downregulation of the 2OG dehydrogenase complex (OGDHc, composing OGDH, DLST, DLD), which converts 2OG to succinyl-CoA (Fig. 2K, L; Supplementary Fig. 2C). Additionally, downstream TCA cycle enzymes (SUCL, SDH, FH) were also suppressed, whereas upstream enzymes (CS and IDH3A/B/G, except ACO1/2) were upregulated (Fig. 2K, M, Supplementary Fig. 2C). Correspondingly, 2OG upstream metabolites citrate and isocitrate levels were elevated, while downstream metabolites succinate, fumarate, and malate were reduced in tumors (Fig. 2H; Supplementary Fig. 2A). Although LDHA and MDH1/2 expressions were decreased, arguing against enhanced L2HG production, L2HGDH was markedly suppressed (Fig. 2K, N, Supplementary Fig. 2C). Importantly, silencing either OGDHc or L2HGDH significantly increased intracellular L2HG, while ectopic expression reversed this effect (Supplementary Fig. 2D–E). Together, these data demonstrate that liver tumors exhibit a metabolic environment that favors L2HG accumulation.

### L2HG sensitizes HCC cells to ferroptosis

**Fig. 3:**
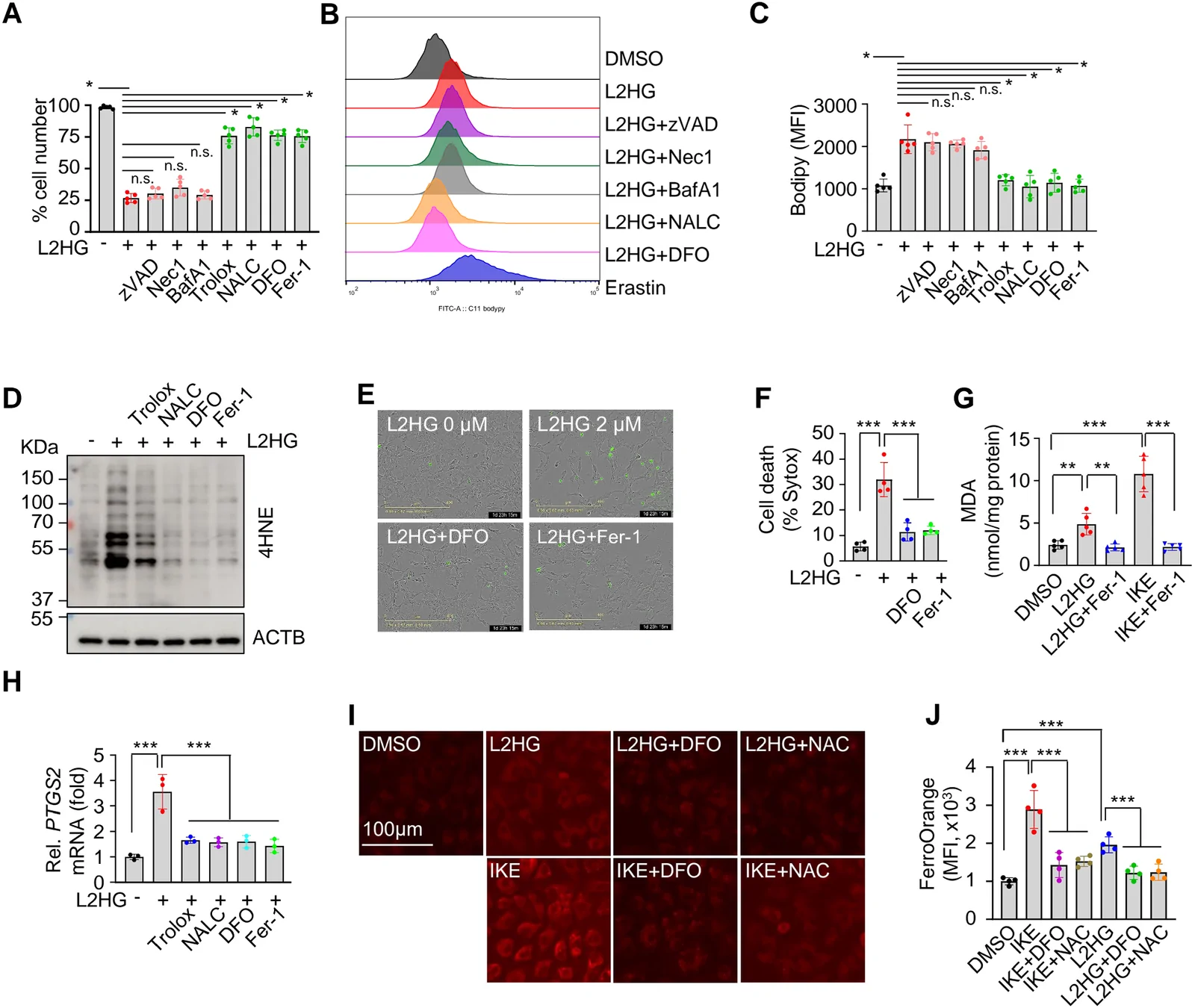
L2HG induces ferroptosis in HCC cells. **A**–**C** Relative cell numbers and lipid peroxidation, assessed by BODIPY-C11 fluorescence, in Hep3B cells treated with 2 mM cell-permeable TFMB–L2HG for 24 h in the presence or absence of inhibitors of apoptosis (zVAD, 50 μM), necroptosis (Nec-1, 10 μM), autophagy (bafilomycin A1, 1 nM), or ferroptosis (Trolox, 100 μM; N-acetylcysteine (NALC), 1 mM; deferoxamine (DFO), 100 μM; ferrostatin-1 (Fer-1), 1 μM). **D**–**G** Induction of lipid peroxidation and iron accumulation in Hep3B cells following TFMB-L2HG treatment (2 mM, 24 h), with or without ferroptosis inhibitors (Trolox, NALC, DFO, and Fer-1). Lipid peroxidation was evaluated by immunoblotting of 4-hydroxynonenal (4-HNE) protein adducts (**D**), cell death determined by Sytox green staining (**E**–**F**) malondialdehyde (MDA) quantification (**G**), and PTGS2 mRNA expression by RT–PCR (**H**). Intracellular ferrous iron (Fe²⁺) levels were measured by FerroOrange staining (**I**), with mean fluorescence intensities quantified (**J**). The data are presented as mean ± SD from different biological experiments (*n* = 3–4). *p < 0.05, **p < 0.01, ***p < 0.001 by unpaired Student’s *t* test.

To directly assess the functional role of L2HG, HCC cells were treated with a cell-permeable trifluoromethylbenzyl ester form of L2HG (TFMB-L2HG). TFMB-L2HG suppressed HCC cell proliferation in a dose-dependent manner (Supplementary Fig. 3A) and induced ferroptosis as evidenced by enhanced sytox green staining (Supplementary Fig. 3B). Evidently, the growth-inhibitory effect of TFMB-L2HG was rescued by ferroptosis inhibitors, including Trolox (a lipophilic antioxidant), N-acetyl-L-cysteine (NALC), deferoxamine (DFO) (an iron chelator), and ferrostatin-1 (Fer-1) (a ferroptosis-specific inhibitor), but not by inhibitors of apoptosis (zVAD-FMK), necroptosis (Necrostatin-1), or autophagy (Bafilomycin A1) (Fig. 3A). These results indicate that TFMB-L2HG-induced cell death is ferroptosis-dependent.

Consistently, TFMB-L2HG increased hallmarks of ferroptosis, including lipid peroxidation as measured by BODIPY-C11 fluorescence (Fig. 3B, C), increased 4-hydroxynonenal (4-HNE) protein adduct formation (Fig. 3D), increased cell death (Fig. 3E, F), elevated malondialdehyde (MDA) levels (Fig. 3G), and upregulation of PTGS2 expression (Fig. 3H). Notably, these effects were markedly attenuated by Fer-1, NAC, and DFO. Furthermore, TFMB-L2HG significantly increased intracellular ferrous iron (Fe^2+^) levels (Fig. 3I, J). Notably, TFMB-L2HG induced ferroptosis across a panel of HCC cell lines (Supplementary Fig. 3C), indicating a general effect. Together, these findings demonstrate that L2HG is sufficient to sensitize HCC cells to ferroptosis.

### L2HG disrupts GSH homeostasis

To elucidate the mechanism by which L2HG promotes ferroptosis, we determined whether L2HG affects lipid metabolism, iron metabolism and coenzyme Q_10_, which were well demonstrated to be involved in ferroptosis regulation [2], for the expression of ACSL4, TFRC, AIFM2 (aka FSP1) and DHODH. Notably, L2HG treatment has no effect on their expression (Supplemental Fig. 4A, B), suggesting their irrelevance to the L2HG-mediated ferroptosis response in liver tumors.

**Fig. 4:**
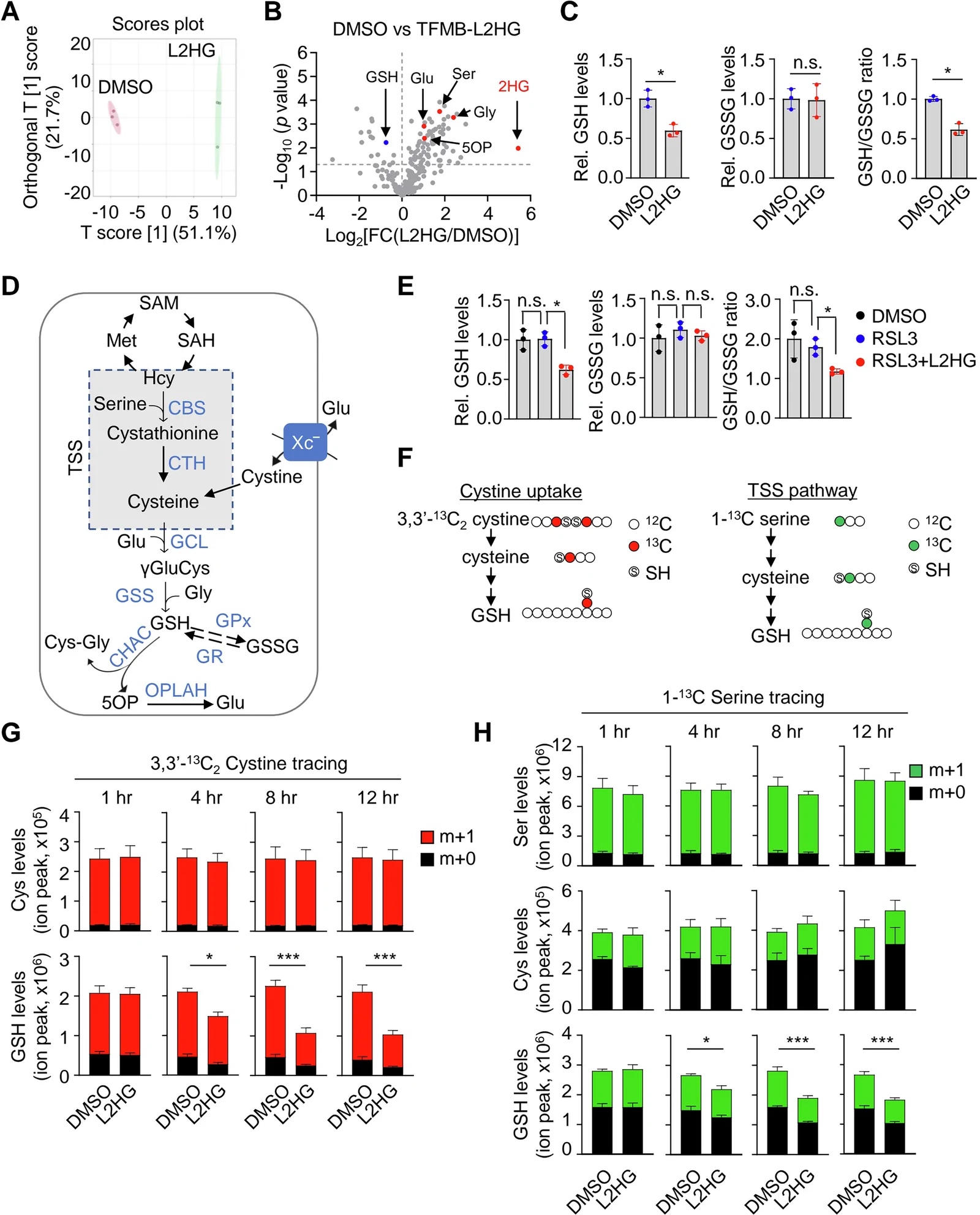
L2HG disrupts GSH homeostasis. LC-MS/MS analysis metabolite profiles of Hep3B cells under TFMB-L2HG treatment (2 mM, 48 h), shown as principal component analysis (PCA) (**A**) and volcano plot analysis (**B**). **C** Ion peak of reduced (GSH), oxidized (GSSG) glutathione, and their ratios following TFMB-L2HG treatments in Hep3B. **D** Schematic epresentation of GSH hemostasis, including GSH biosynthesis from cysteine (derived from cystine uptake and de novo cysteine synthesis via the methionine cycle through the transsulfuration pathway [TSS]), GSH utilization to form GSSG, and GSH degradation to 5-oxoproline (5OP) and cysteinylglycine (Cys-Gly). Enzymes involved in each step are indicated in blue. Met, methionine; SAM, S-Adenosyl methionine; SAH, S-adenosylhomocysteine; Hcy, homocysteine. **E** Effects of ferroptosis induction by RSL3 (200 nM) and TFMB-L2HG (2 mM) on intracellular GSH and GSSG levels and the GSH/GSSG ratio. **F** Schematic of stable isotope tracing of cystine and serine into GSH. **G**, **H** Quantification of incorporation of 3,3’-^13^C_2_ cystine (100 μM) or 1-^13^C serine into cysteine and subsequently into GSH via cystine uptake or the transsulfuration pathway, respectively, in Hep3B cells treated with TFMB–L2HG (2 mM). Ser, serine; Cys, cysteine. The data is presented as mean ± SD from biological experiments (*n* = 3-4). *p < 0.05.

We next performed untargeted LC-MS/MS metabolomic profiling in Hep3B cells treated with TFMB-L2HG (Fig. 4A, B; Supplementary Fig. 4C; Supplemental Table 8). Notably, TFMB-L2HG significantly reduced intracellular GSH levels and decreased the GSH/GSSG ratio (Fig. 4C). Consistently, L2HGDH knockout reduced, whereas L2HGDH^OPT^ expression increased, GSH levels in Hep3B cells (Supplementary Fig. 4D–E), confirming that the L2HG/L2HGDH axis regulates GSH homeostasis.

GSH levels are controlled by synthesis (with cysteine as a critical precursor), utilization (by GPXs to form GSSG and present antioxidative effect), and degradation (to 5-oxoproline, 5OP, which is further degraded to glutamate by 5-oxoprolinase, OPLAH) (Fig. 4D). We first examined whether L2HG affects GSH utilization for the expression and activity regulation of GPX4, the main glutathione peroxidase in the regulation of ferroptosis. Notably, GPX4 expressions at both mRNA and protein levels were not affected by TFMB-L2HG (Supplementary Fig. B, F). Additionally, under the cotreatment of RSL3 to suppress GPX4-mediated GSH utilization, we still detected that TFMB-L2HG can reduce GSH levels (Fig. 4E). More importantly, because enhanced GSH utilization would be expected to counteract ferroptosis, these findings suggest that increased GSH consumption is unlikely to account for the observed GSH depletion. Thus, we speculated that the ferroptosis induced by L2HG may be initiated from suppressed GSH biosynthesis, accelerated degradation or both.

We also evaluated whether impaired cystine uptake or transsulfuration (TSS) contributes to GSH loss. Surprisingly, TFMB-L2HG moderately increased cystine uptake (Supplementary Fig. 4G). Notably, unlike most cell types that depend heavily on extracellular cystine for uptake, hepatocytes derive approximately 50% of the intracellular cysteine via the transsulfuration (TSS) pathway from methionine (Fig. 4D) [30]. We revisited the LC-MS/MS results and found that the level of cystathionine, a crucial intermediate of TSS, is slightly but insignificantly increased (Supplementary Fig. 4H).

To further affirm L2HG shows no effect on cystine uptake or TSS pathway activities for GSH level regulation, we assessed the metabolism of isotype 3,3’-^13^C_2_ cystine and 1-^13^C serine separately (Fig. 4F). No ^13^C labeled GSH change was observed at 1 h of tracing, indicating that GSH synthesis capacity was intact. However, significantly reduced levels of ^13^C labeled GSH were detected after 4 h of tracing, while continuous tracing to 12 h make the difference even augmented (Fig. 4G, H). Notably, the expression of factors involved in the synthesis of GSH, GCLC, GCLM, and GSS, were either upregulated or not changed under TFMB-L2HG treatment (Supplementary Fig. 4F). Additionally, there are increased levels of glycine and glutamate, substrates for the GSH synthesis (Fig. 4B; Supplementary Fig. 4C,H). Together, these findings suggest that L2HG-induced GSH depletion does not result from impaired synthesis, cystine uptake, TSS activity, or enhanced utilization, but instead suggests accelerated GSH degradation.

We subsequently re-examined the metabolomic data, from which we detected a marked accumulation of 5-oxoproline (5OP), the direct product of GSH degradation, and glutamate (a following degradation product of 5OP) (Fig. 4B; Supplementary Fig. 4C), consistent with enhanced 5OP turnover. Notably, glutamate has been shown to be involved in the cystine uptake, GSH biosynthesis and GSH degradation for the regulation of ferroptosis (Fig. 4D). Additionally, since other metabolic pathways such as glutaminolysis also contribute to the production of glutamate, we can’t affirm the increase of glutamate is solely from 5OP degradation. To minimize the effect of L2HG on glutamine/glutamate metabolism, we measured GSH, 5OP and glutamate levels in the presence of L2HG and CB839, an inhibitor of glutaminolysis. CB-839 alone reduced glutamate and GSH levels, and co-treatment with TFMB-L2HG significantly increased 5OP and glutamate while further depleting GSH (Supplementary Fig. 4I), supporting enhanced GSH degradation.

To determine whether L2HGDH expression changes similarly affect GSH degradation, we measured 5OP levels in sg*L2HGDH* and L2HGDH^OPT^ Hep3B cells. sg*L2HGDH* was found to significantly increase while L2HGDH^OPT^ reduced 5OP levels (Supplementary Fig. 4J–K), which reinforced a link between L2HGDH/L2HG regulation and GSH degradation. Together, these data indicated that L2HG regulates ferroptosis through enhanced GSH degradation.

### L2HG promotes GSH degradation via the ATF3/CHAC1 axis

To reveal the mechanism of L2HG on GSH degradation, we perturbed the function of L2HG as a structural analog of 2OG, to modulate the activity of 2OG-dependent dioxygenases (2OGDDs) such as JmjC domain-containing histone demethylases in affecting chromatin structures (Supplementary Fig. 5A). Indeed, we observed markedly elevated global methylation levels in L2HGDH^low^ compared to L2HGDH^high^ cells (Supplementary Fig. 5B–C). In addition, L2HGDH^OPT^ reduced, while sg*L2HGDH* and TFMB-L2HG significantly increased histone methylation (Supplementary Fig. 5D–F), supporting the role of the L2HG/L2HGDH axis on histone methylation regulation. Notably, this L2HG-induced histone hypermethylation is unrelated to the expression changes of histone demethylases (KDMs) or methyltransferases (KMTs), since no single enzyme class was dominantly affected (Supplementary Fig. 5G). In addition, selected expression modulation for histone demethylase KDM6B (by sh*KDM6B*), the most affected KDM under L2HG treatment, presents no effect on the expression of CHAC1 (Supplementary Fig. 5H). These observations indicate that L2HG modulates histone methylation for the activity of 2OGDDs through competing with the cofactor 2OG.

**Fig. 5:**
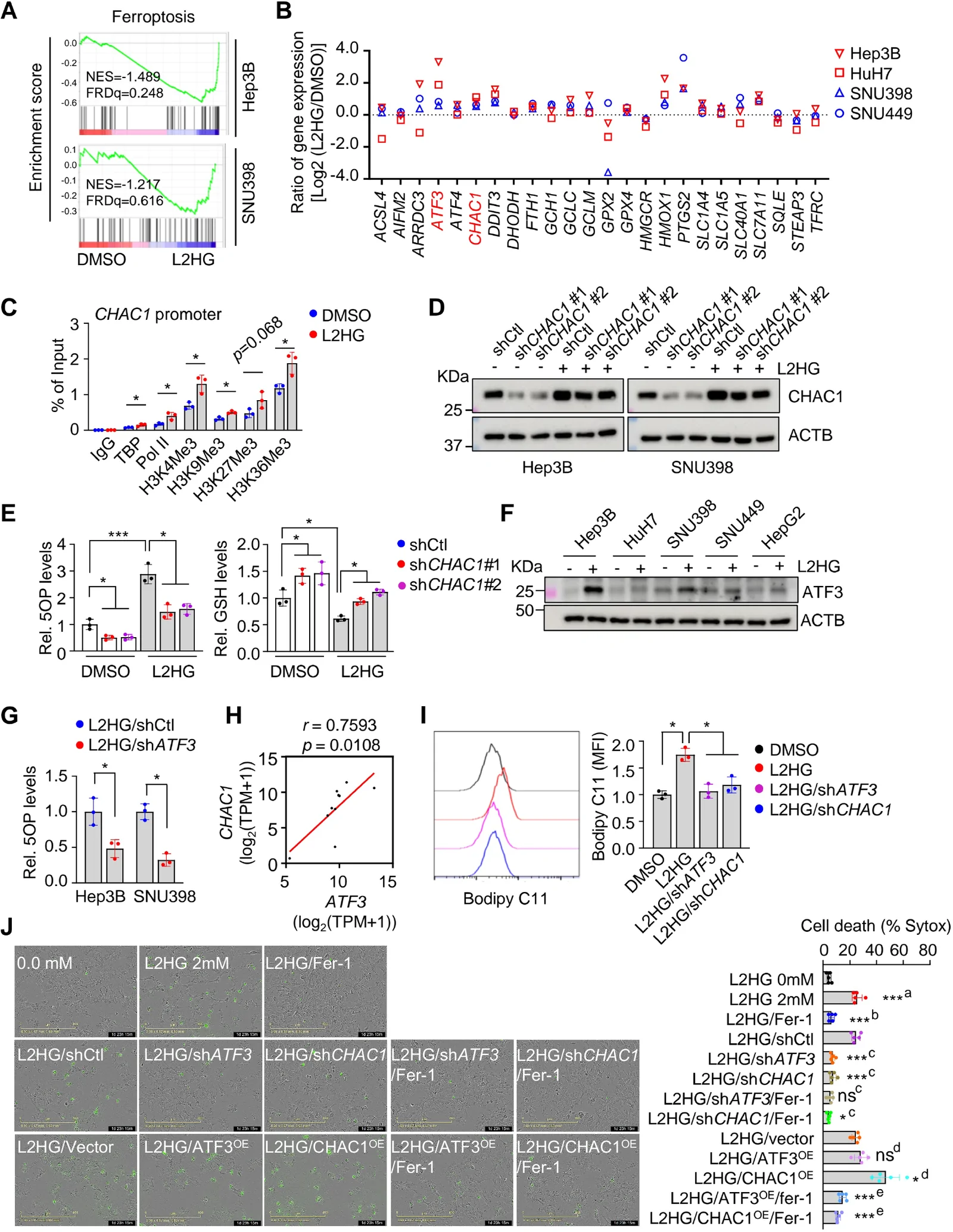
L2HG promotes glutathione degradation to 5-oxoproline through the ATF3/CHAC1 axis. **A** Transcriptional pathway analysis of ferroptosis-related genes in L2HGDH^high^ (Hep3B) and L2HGDH^low^ (SNU398) HCC cell lines following TFMB-L2HG treatment. **B** Relative expression of ferroptosis-associated genes in Hep3B, HuH7, SNU398 and SNU449 cells treated with TFMB-L2HG compared with DMSO control, shown as log_2_[fold change (L2HG/DMSO)]. Note, not all genes are expressed in all four HCC lines, such as *PTGS2* is not expressed in HuH7. **C** Chromatin immunoprecipitation (ChIP) analysis of TATA-binding protein (TBP), RNA polymerase II (Pol II), and histone methylation markers at the *CHAC1* promoter in Hep3B cells. **D** Immunoblot analysis of *CHAC1* knockdown using two independent shRNA constructs (sh*CHAC1* #1 and #2) in Hep3B and SNU398 cells under TFMB-L2HG treatment. **E** Relative intracellular 5-oxoproline (5OP) and GSH levels in sh*CHAC1*-expressing Hep3B and SNU398 cells treated with TFMB-L2HG. **F** Immunoblot analysis of ATF3 protein expression in multiple HCC cell lines following TFMB-L2HG treatment (2 mM). **G** Relative 5OP levels in Hep3B and SNU398 cells following ATF3 knockdown (sh*ATF3*). **H** Correlation analysis of CHAC1 and ATF3 mRNA expression in 10 HCC cell lines stratified as L2HGDH^high^ (HepG2, PLC/PRF/5, HuH7, Hep3B) or L2HGDH^low^ (SNU423, SNU398, SNU449, SNU475, SK-HEP1) using RNA-seq data from the CCLE dataset. **I** Lipid peroxidation assessed by BODIPY-C11 staining in Hep3B cells treated with TFMB-L2HG, sh*ATF3*, or sh*CHAC1*, with relative mean fluorescence intensity (MFI) quantified. **J** Incucyte Sytox green staining determined the cell death induced by TFMB-L2HG treatment, and its rescue by Fer-1. The ferroptotic cell death under the silence of ATF3 and CHAC1 by shRNA (sh*ATF3* and sh*CHAC1*) or ectopic overexpression (ATF3^OE^ and CHAC1^OE^) and their rescue by Fer-1 were determined after 48 h of culture. The data is presented as mean ± SD of 3 different biological experiments. *p < 0.05. a-e, statistical analysis was conducted against no L2HG treatment (a), L2HG 2 mM treatment (b), shCtl treatment (c), empty vector treatment (d) or their overexpression (e).

We next performed transcriptomic analysis in Hep3B, HuH7, SNU398, and SNU449 cells to identify potential factors that are regulated by L2HG for GSH degradation. RNA-seq results showed that TFMB-L2HG impacted ferroptosis signals (Fig. 5A). Notably, the expression of CHAC1, a glutathione-specific gamma-glutamylcyclotransferase, which mediates GSH degradation to 5OP, was elevated (Fig. 5B).

To affirm the global histone methylation changes influence CHAC1 transcription, we examined histone modifications by ChIP analysis. Increased enrichment of both activating (H3K4me3) and repressive (H3K9me3, H3K27me3) histone marks was detected at the CHAC1 promoter, accompanied by enhanced recruitment of TATA-binding protein and RNA polymerase II (Fig. 5C), indicating transcriptional activation within a bivalent chromatin context. To further support that chromatin structure changes under TFMB-L2HG treatment promote the expression of CHAC1, we conducted ATAC-seq analysis. This revealed that TFMB-L2HG increased chromatin accessibility in CHAC1 locus (Supplementary Fig. 5I), supporting a transcriptional activation mechanism.

To demonstrate that CHAC1 is responsible for L2HG-mediated GSH degradation, we manipulated CHAC1 expression. Remarkably, *CHAC1* silencing (sh*CHAC1*) restored GSH levels and significantly reduced 5OP accumulation following TFMB-L2HG treatment in both Hep3B and SNU398 cells (Fig. 5D, E; Supplementary Fig. 6A), demonstrating that CHAC1 mediates L2HG-induced GSH degradation.

To further gain insight into the mechanism of L2HG on CHAC1 regulation, we probed the transcription factors involved. CHAC1 has been reported under transcriptional regulation by ATF3 and ATF4 via a bipartite ATF/CRE response element under stress response in atherosclerosis [31]. We also recently showed that ATF3 can upregulate CHAC1 expression in retinal pigment epithelial cells [32, 33]. Interestingly, though we did not detect an effect on the expression of ATF4, we observed that TFMB-L2HG increased ATF3 expression (Fig. 5B, F). ATF3 silencing (sh*ATF3*) was found to suppress the expression of CHAC1 (Supplementary Fig. 6B), along with other known ATF3 targets such as PTGS2, HMOX1, DDIT3 and TFRC [31–33]. Consistently, ATAC-seq results also showed that chromatin at ATF3 and these downstream target gene loci were more accessible under TFMB-L2HG treatment (Supplementary Fig. 5I). As further evidence for the regulation of CHAC1 by L2HG through ATF3, we observed that the induction effect of L2HG on CHAC1 expression was diminished by sh*ATF3* (Supplementary Fig. 6C), and the binding of ATF3 to CHAC1 promoter was significantly increased by L2HG (Supplementary Fig. 6D). In consistent, CHAC1 promoter activity assay also demonstrated that L2HG and sg*L2HGDH* treatment significantly induced CHAC1 promoter activities (Supplementary Fig. 6E), whereas the induction activity of ATF3 on CHAC1 expression was demolished by sh*ATF3* or promoter ATF/CRE motif mutation (Supplementary Fig. 6E). These observations support that L2HG induces CHAC1 promoter activities through ATF3 binding to the promoter ATF/CRE motif.

Most importantly, in line with CHAC1’s function in catalyzing GSH degradation to 5OP, sh*ATF3* was found to significantly decrease 5OP levels (Fig. 5G). Consistent with these findings, ATF3 and CHAC1 expression levels were positively correlated in CCLE HCC cell lines and TCGA-LIHC patient samples (Fig. 5H; Supplementary Fig. 6F), providing additional support for ATF3 as a key transcriptional regulator of CHAC1 in HCC.

We lastly determined whether expression changes of ATF3/CHAC1 will affect L2HG-induced ferroptosis. Interestingly, the suppression effect of L2HG on cell proliferation was diminished by sh*ATF3* and sh*CHAC1* in both Hep3B and SNU398 cells (Supplementary Fig. 6G). The ferroptosis signals as measured by Bodipy C11 fluorescence and Sytox green staining were also suppressed. In contrast, overexpression of ATF3 and CHAC1 significantly increased ferroptosis signals (Fig. 5I–J; Supplementary Fig. 6H). Together, these findings delineate the regulatory mechanism by which L2HG modulates histone methylation and thus activates the ATF3/CHAC1 signaling to promote GSH degradation and sensitize cells to ferroptosis.

### Targeting L2HGDH induces ferroptosis in vivo

**Fig. 6:**
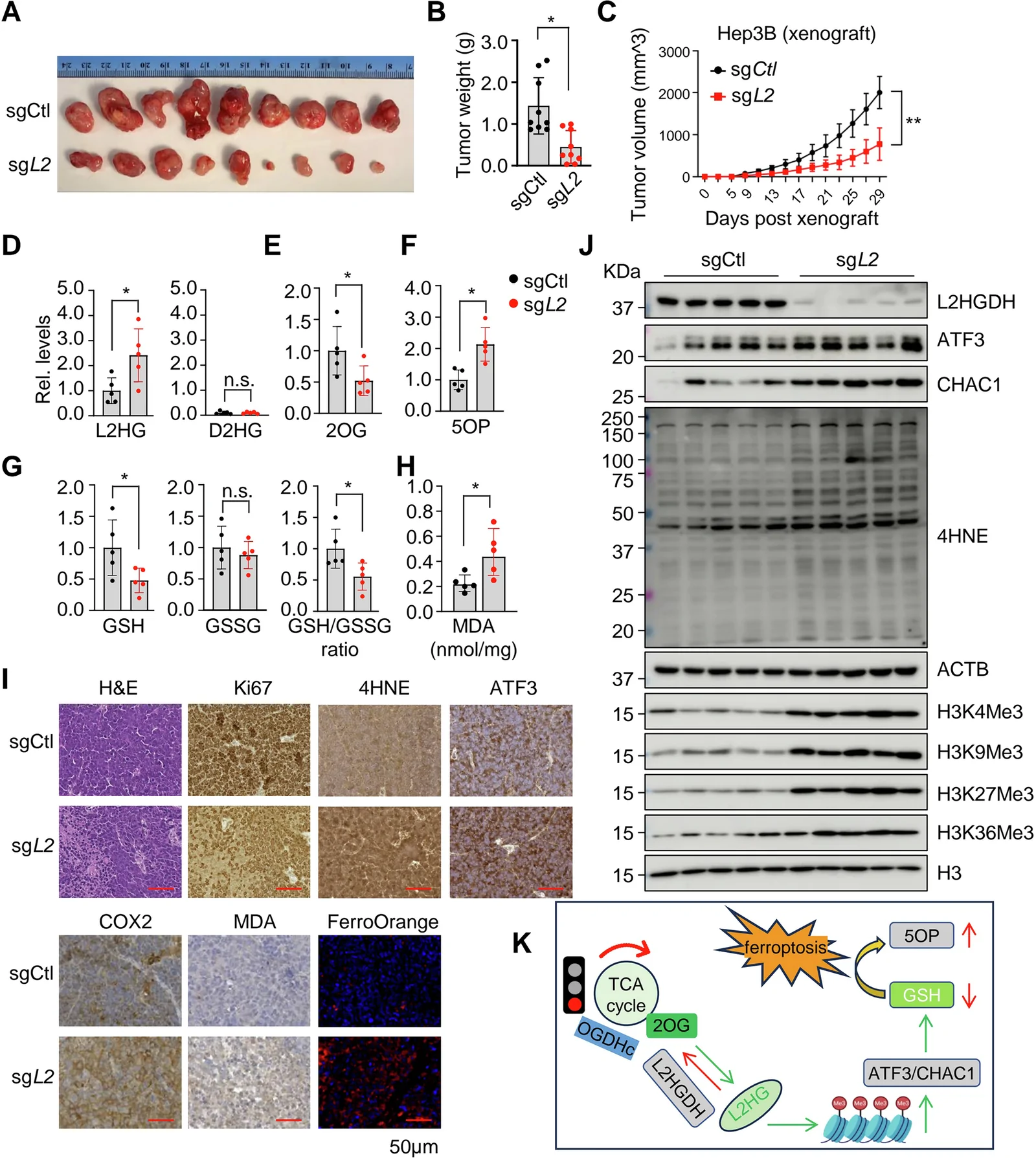
*L2HGDH* silencing suppresses tumor growth and promotes ferroptosis in vivo. Representative images (**A**), tumor weights (**B**), and tumor volumes (**C**) of sgControl and sg*L2HGDH* Hep3B cell-derived xenografts established in NSG mice. **D**–**H** Metabolomic analyses of sgControl and sg*L2HGDH* Hep3B tumor xenografts showing intracellular levels of L2HG and D2HG (**D**), 2-oxoglutarate (2OG) (**E**), 5-oxoproline (5OP) (**F**), and reduced glutathione (GSH), oxidized glutathione (GSSG), and the GSH/GSSG ratio (**G**), or tissue MDA levels (**H**). **I** Immunohistochemical analysis of sgControl and sg*L2HGDH* Hep3B tumor xenograft sections. **J** Immunoblot analysis of protein extracts from sgControl and sg*L2HGDH* Hep3B tumor xenografts. The data is presented as mean ± SD (*n* = 5–9). *p < 0.05, **p < 0.01. **K** Schematic model illustrating the mechanism of ferroptosis induction by L2HG. A metabolic environment (reduced L2HGDH expression and suppressed TCA cycle activity) prone to L2HG accumulation in HCC. L2HG increases ATF3/CHAC1 expression through histone hypermethylation to promote GSH degradation to 5-oxoproline (5OP) and induces ferroptosis.

We next sought to determine whether L2HGDH inhibition to increase L2HG levels will sensitize cancer cells to ferroptosis in vivo. The sg*L2HGDH* Hep3B HCC cells were subcutaneously implanted into NSG mice for tumor growth. Compared to sgRNA control, sg*L2HGDH* suppressed effectively the growth of Hep3B xenografts, by reduced tumor weight and volume (Fig. 6A–C). In agreement with the function of L2HGDH in mediate L2HG conversion to 2OG, we detected significantly increased levels of L2HG, accompanied with reduced 2OG levels in sg*L2HGDH* xenografts (Fig. 6D, E).

Consistent with enhanced ferroptosis by L2HG, we detected reduced GSH but increased 5OP levels in sg*L2HGDH* xenografts, whereas lipid peroxidation detection showed increased malondialdehyde levels (Fig. 6F–H). Notably, sg*L2HGDH* tumors exhibited reduced Ki67 staining, increased 4HNE accumulation, elevated ATF3 and CHAC1 expression, and increased histone methylation (Fig. 6I, J). In addition, in Hep3B sg*L2HGDH* xenografts, we observed significantly increased staining of COX2, MDA and ferrous iron (Fe2 + ) staining (Fig. 6I). Together, these in vitro and in vivo findings demonstrate that L2HG/L2HGDH presents ferroptosis regulatory functions through affecting 2OGDDs activities, leading to histone hypermethylation in the promoters of the ATF3 and CHAC1. Together, elevated levels of L2HG led to enhanced GSH degradation to 5OP and interrupted GSH hemostasis to sensitize HCC cells to ferroptosis (Fig. 6K).

## Discussion

Ferroptosis has emerged as a central therapeutical vulnerability in HCC. Canonical regulation of ferroptosis has been largely defined by GSH synthesis through cystine import via the xcˉ system composed of the light chain xCT (SLC7A11) and heavy chain 4F2hc (SLC3A2) and its utilization by GPX4, which together constitute two major ferroptosis checkpoints. However, accumulating evidence has identified multiple GPX4- and SLC7A11-independent regulatory pathways [34], including FSP1/CoQ10, [35] P53/lipoxygenase signaling [36], DHODH/ubiquinone [37], GCH1/tetrahydrobiopterin, [6] and SCD1-monounsaturated fatty acid (MUFA) synthesis[38]. Sex hormones have also been shown to modulate ferroptotic sensitivity through phospholipid remodeling via MBOAT1/2 in a GPX4-independent manner[39]. Despite these advances, the endogenous metabolic pathways governing ferroptosis sensitivity in liver tumors remain incompletely understood. Here, through unbiased CRISPRa screening, patient-derived metabolomic analyses, and mechanistic interrogation, we identify L2HG metabolism as a previously unrecognized ferroptosis checkpoint in HCC. We establish L2HGDH as a critical metabolic safeguard that suppresses ferroptotic cell death. Mechanistically, pathological accumulation of L2HG, rather than alterations in canonical lipid peroxidation or iron metabolism, initiates ferroptosis by epigenetically accelerating glutathione degradation via an ATF3/CHAC1-dependent pathway. Together, these findings uncover L2HG metabolism as a key determinant of ferroptotic vulnerability in liver tumors.

Previously, L2HGDH was shown to reduce lipid peroxidation and protect against ferroptosis [6, 40]. Consistently, our study demonstrates that L2HGDH promotes liver tumor proliferation. Similarly, L2HGDH-deficient mice showed increased apoptosis and cell death[41]. In contrast, L2HGDH appears to play an opposing role in colorectal cancer [42] and clear cell renal cell carcinoma [43, 44], in which L2HGDH suppresses tumor growth, while its silencing enhances proliferation. Additionally, in cerebral organoids, L2HGDH silencing led to excessive growth through MYC upregulation [45], whereas in B cell lymphomas, MYC was found to regulate L2HGDH expression without affecting cell proliferation[46]. These contradictory observations suggest that L2HGDH displays a context-dependent role across different tissue types and microenvironments.

Although L2HGDH catalyzes the conversion of L2HG back to 2OG, the mechanisms underlying L2HG production remain poorly defined. It is thought to arise primarily from the promiscuous enzymatic activities of LDHA and MDH1/2[27–29]. Additionally, the availability of 2OG also influences intracellular L2HG levels[27]. In colorectal cancer, altered expression of the OGDHc, particularly OGDH and DLST, has been implicated in L2HG accumulation. In our study, we observed a significant downregulation of OGDHc in tumors compared to adjacent normal liver tissues. Furthermore, changes in the expression of TCA cycle enzymes support a broader metabolic shift that favors L2HG buildup. Our findings suggest that the metabolic environment in liver tumors promotes L2HG accumulation.

Importantly, L2HG was found to sensitize HCC cells to ferroptosis, highlighting its critical role in influencing liver tumor cell survival. Previous studies showed both enantiomers, D2HG and L2HG, can promote ferroptosis, reduce cell viability, and increase lipid peroxidation in human squamous cell carcinoma cells[47]. Mechanistically, D2HG and L2HG were also reported to exert broad anti-proliferative effects and reduce viability by suppressing the FTO/m6A/MYC signaling[48]. FTO inhibition has been linked to enhanced ferroptosis through upregulation of ACSL4 and TFRC [49] and downregulation of SLC7A11 and GPX4, [50] implicating a ferroptosis regulatory effect of D2HG and L2HG. Moreover, L2HG accumulation, induced by OGDH inhibition and FOXO1 activation, was shown to inhibit endothelial cell proliferation, promote quiescence, and suppress angiogenesis[51]. Additionally, L2HG increases dependence on exogenous serine to support proliferation, redox balance, and tumor growth via KDM6A-mediated H3K27me3 regulation[44]. Our recent findings also demonstrate that L2HG disrupts NRF2 binding to antioxidant genes, thereby promoting ferroptosis sensitivity[20]. Conversely, L2HG was also reported to support tumor cell growth through activation of the mTOR-ATF4 axis and amino acid metabolism [42], modulate RNA methylation to influence DNA/RNA damage repair, [52] and interfere with HIF-driven transcriptomic reprogramming[53]. These observations, including ours, underscore the context-dependent multifaceted impacts of L2HG on tumor growth and survival.

Mechanistically, our studies showed L2HG stimulates non-canonical ferroptosis mechanism driven by glutathione degradation through ATF3/CHAC1 induction, ultimately resulting in a net decrease in intracellular GSH levels. L2HG and D2HG are known to act as competitive inhibitors of 2OG and modulate the activity of 2OGDDs, for DNA and histone methylation regulation, thereby affecting gene expression through epigenetic mechanisms[54]. Our findings revealed L2HG increases histone methylation and chromatin structures to affect the expression of ATF3/CHAC1 for ferroptosis regulation. Previous studies have shown that L2HG rewires metabolism of cystine, glycine, serine, and glutamine, key substrates for GSH biosynthesis[42, 43]. Additionally, we observed elevated 5OP levels following L2HG treatment, consistent with increased GSH turnover. Importantly, previous investigations also revealed that L2HG can enhance ATF4 mRNA stability and translation and thus lead to increased CHAC1 expression and GSH degradation[42]. However, in a different cellular context in clear cell renal cell carcinoma, L2HG was shown to suppress ATF4 expression through an as-yet-unknown mechanism, resulting in decreased expression of amino acid biosynthesis genes[43]. These reports in distinct tumor types and our observations in HCC suggest that L2HG exerts differential effects on amino acid metabolism and ferroptosis susceptibility in a tumor environment dependent manner.

## Supplementary information

Original data

Supplemental Figures

Supplemental Table 1. Human patient samples

Supplementary Table 2. DNA oligoes

Supplementary Table 3. Customized HPLM medium

Supplementary Table 4. List of plasmids

Supplementary Table 5. Antibodies

Supplementary Table 6. Metabolic library CRISPRa sgRNA screen data

Supplementary Table 7. Metabolite profile (human patient, NT vs T)

Supplementary Table 8. Metabolite profile (DMSO vs TFMB-L2HG, Hep3B)

## Data Availability

Public datasets used in this study were downloaded from the TCGA portal (https://portal.gdc.cancer.gov/), Human Protein Atlas (https://www.proteinatlas.org/) and the Cancer Cell Line Encyclopedia (CCLE) (https://sites.broadinstitute.org/ccle/). RNA-seq, ATAC-seq and metabolic screen data have been deposited at Gene Expression Omnibus under the accessions GSE302217 and GSE301997. The remaining data supporting the findings of this paper are provided with supplementary information.

## References

[1] Tang D, Chen X, Kang R, Kroemer G. Ferroptosis: molecular mechanisms and health implications. Cell Res. 2021;31:107–25. 10.1038/s41422-020-00441-1 PMC8026611 33268902

[2] Stockwell BR. Ferroptosis turns 10: emerging mechanisms, physiological functions, and therapeutic applications. Cell. 2022;185:2401–21. 10.1016/j.cell.2022.06.003 PMC9273022 35803244

[3] Lei G, Zhuang L, Gan B. Targeting ferroptosis as a vulnerability in cancer. Nat Rev Cancer. 2022;22:381–96. 10.1038/s41568-022-00459-0 PMC10243716 35338310

[4] Dixon SJ, Stockwell BR. The hallmarks of ferroptosis. Annu Rev Canc Biol. 2019;3:35–54. 10.1146/annurev-cancerbio-030518-055844 PMC12851585 41613499

[5] Wu Y, Li H, Yue K, Jing C, Duan Y. Ferroptosis in cancer: metabolism, mechanisms and therapeutic prospects. Mol Cancer. 2025;24:303. 10.1186/s12943-025-02520-6 PMC12667084 41327191

[6] Kraft VAN, Bezjian CT, Pfeiffer S, Ringelstetter L, Muller C, Zandkarimi F, et al. GTP Cyclohydrolase 1/tetrahydrobiopterin counteract ferroptosis through lipid remodeling. ACS Cent Sci. 2020;6:41–53. 10.1021/acscentsci.9b01063 PMC6978838 31989025

[7] Kang YP, Mockabee-Macias A, Jiang C, Falzone A, Prieto-Farigua N, Stone E, et al. Non-canonical glutamate-cysteine ligase activity protects against ferroptosis. Cell Metab. 2021;33:174–89.e7. 10.1016/j.cmet.2020.12.007 PMC7839835 33357455

[8] Fujihara KM, Zhang BZ, Jackson TD, Ogunkola MO, Nijagal B, Milne JV, et al. Eprenetapopt triggers ferroptosis, inhibits NFS1 cysteine desulfurase, and synergizes with serine and glycine dietary restriction. Sci Adv. 2022;8:eabm9427. 10.1126/sciadv.abm9427 PMC9473576 36103522

[9] Zhu J, Berisa M, Schworer S, Qin W, Cross JR, Thompson CB. Transsulfuration activity can support cell growth upon extracellular cysteine limitation. Cell Metab. 2019;30:865–76.e5. 10.1016/j.cmet.2019.09.009 PMC6961654 31607565

[10] Cao J, Chen X, Jiang L, Lu B, Yuan M, Zhu D, et al. DJ-1 suppresses ferroptosis through preserving the activity of S-adenosyl homocysteine hydrolase. Nat Commun. 2020;11:1251. 10.1038/s41467-020-15109-y PMC7060199 32144268

[11] Tsurusaki S, Tsuchiya Y, Koumura T, Nakasone M, Sakamoto T, Matsuoka M, et al. Hepatic ferroptosis plays an important role as the trigger for initiating inflammation in nonalcoholic steatohepatitis. Cell Death Dis. 2019;10:449. 10.1038/s41419-019-1678-y PMC6579767 31209199

[12] Yu Y, Jiang L, Wang H, Shen Z, Cheng Q, Zhang P, et al. Hepatic transferrin plays a role in systemic iron homeostasis and liver ferroptosis. Blood. 2020;136:726–39. 10.1182/blood.2019002907 PMC7414596 32374849

[13] Chen J, Li X, Ge C, Min J, Wang F. The multifaceted role of ferroptosis in liver disease. Cell Death Differ. 2022;29:467–80. 10.1038/s41418-022-00941-0 PMC8901678 35075250

[14] Escuder-Rodriguez JJ, Liang D, Jiang X, Sinicrope FA. Ferroptosis: biology and role in gastrointestinal disease. Gastroenterology. 2024;167:231–49. 10.1053/j.gastro.2024.01.051 PMC11193643 38431204

[15] Cantor JR, Abu-Remaileh M, Kanarek N, Freinkman E, Gao X, Louissaint A Jr., et al. Physiologic medium rewires cellular metabolism and reveals uric acid as an endogenous inhibitor of UMP synthase. Cell. 2017;169:258–72.e17. 10.1016/j.cell.2017.03.023 PMC5421364 28388410

[16] Unlu G, Prizer B, Erdal R, Yeh HW, Bayraktar EC, Birsoy K. Metabolic-scale gene activation screens identify SLCO2B1 as a heme transporter that enhances cellular iron availability. Mol Cell. 2022;82:2832–43.e7. 10.1016/j.molcel.2022.05.024 PMC9356996 35714613

[17] Uhlen M, Oksvold P, Fagerberg L, Lundberg E, Jonasson K, Forsberg M, et al. Towards a knowledge-based human protein atlas. Nat Biotechnol. 2010;28:1248–50. 10.1038/nbt1210-1248 21139605

[18] Inde Z, Rodencal J, Dixon SJ. Quantification of drug-induced fractional killing using high-throughput microscopy. STAR Protoc. 2021;2:100300. 10.1016/j.xpro.2021.100300 PMC7829341 33532743

[19] Xi C, Pang J, Xue W, Cui Y, Jiang N, Zhi W, et al. Transsulfuration pathway activation attenuates oxidative stress and ferroptosis in sickle primary erythroblasts and transgenic mice. Commun Biol. 2025;8:15. 10.1038/s42003-024-07424-7 PMC11704341 39762627

[20] Xi C, Pang J, Zhi W, Chang CS, Siddaramappa U, Shi H, et al. Nrf2 sensitizes ferroptosis through l-2-hydroxyglutarate-mediated chromatin modifications in sickle cell disease. Blood. 2023;142:382–96. 10.1182/blood.2022018159 PMC10485372 37267508

[21] Chavez A, Scheiman J, Vora S, Pruitt BW, Tuttle M, Iyer PR, et al. Highly efficient Cas9-mediated transcriptional programming. Nat Methods. 2015;12:326–8. 10.1038/nmeth.3312 PMC4393883 25730490

[22] Huang Q, Tan Y, Yin P, Ye G, Gao P, Lu X, et al. Metabolic characterization of hepatocellular carcinoma using nontargeted tissue metabolomics. Cancer Res. 2013;73:4992–5002. 10.1158/0008-5472.CAN-13-0308 23824744

[23] Ferrarini A, Di Poto C, He S, Tu C, Varghese RS, Kara Balla A, et al. Metabolomic analysis of liver tissues for characterization of hepatocellular carcinoma. J Proteome Res. 2019;18:3067–76. 10.1021/acs.jproteome.9b00185 PMC6677583 31188000

[24] Hou G, Ding D, Tian T, Dong W, Sun D, Liu G, et al. Metabolomics-based classification reveals subtypes of hepatocellular carcinoma. Mol Carcinog. 2022;61:989–1001. 10.1002/mc.23455 36121331

[25] Missiaen R, Anderson NM, Kim LC, Nance B, Burrows M, Skuli N, et al. GCN2 inhibition sensitizes arginine-deprived hepatocellular carcinoma cells to senolytic treatment. Cell Metab. 2022;34:1151–67.e7. 10.1016/j.cmet.2022.06.010 PMC9357184 35839757

[26] Mossmann D, Muller C, Park S, Ryback B, Colombi M, Ritter N, et al. Arginine reprograms metabolism in liver cancer via RBM39. Cell. 2023;186:5068–83.e23. 10.1016/j.cell.2023.09.011 PMC10642370 37804830

[27] Intlekofer AM, Wang B, Liu H, Shah H, Carmona-Fontaine C, Rustenburg AS, et al. L-2-Hydroxyglutarate production arises from noncanonical enzyme function at acidic pH. Nat Chem Biol. 2017;13:494–500. 10.1038/nchembio.2307 PMC5516644 28263965

[28] Intlekofer AM, Dematteo RG, Venneti S, Finley LW, Lu C, Judkins AR, et al. Hypoxia induces production of L-2-hydroxyglutarate. Cell Metab. 2015;22:304–11. 10.1016/j.cmet.2015.06.023 PMC4527873 26212717

[29] Oldham WM, Clish CB, Yang Y, Loscalzo J. Hypoxia-mediated increases in L-2-hydroxyglutarate coordinate the metabolic response to reductive stress. Cell Metab. 2015;22:291–303. 10.1016/j.cmet.2015.06.021 PMC4526408 26212716

[30] Mosharov E, Cranford MR, Banerjee R. The quantitatively important relationship between homocysteine metabolism and glutathione synthesis by the transsulfuration pathway and its regulation by redox changes. Biochemistry. 2000;39:13005–11. 10.1021/bi001088w 11041866

[31] Crawford RR, Prescott ET, Sylvester CF, Higdon AN, Shan J, Kilberg MS, et al. Human CHAC1 protein degrades glutathione, and mRNA induction is regulated by the transcription factors ATF4 and ATF3 and a bipartite ATF/CRE regulatory element. J Biol Chem. 2015;290:15878–91. 10.1074/jbc.M114.635144 PMC4505494 25931127

[32] Li X, Gracilla D, Cai L, Zhang M, Yu X, Chen X, et al. ATF3 promotes the serine synthesis pathway and tumor growth under dietary serine restriction. Cell Rep. 2021;36:109706. 10.1016/j.celrep.2021.109706 PMC8491098 34551291

[33] Wang L, Liu Y, Du T, Yang H, Lei L, Guo M, et al. ATF3 promotes erastin-induced ferroptosis by suppressing system Xc. Cell Death Differ. 2020;27:662–75. 10.1038/s41418-019-0380-z PMC7206049 31273299

[34] Ma T, Du J, Zhang Y, Wang Y, Wang B, Zhang T. GPX4-independent ferroptosis-a new strategy in disease’s therapy. Cell Death Discov. 2022;8:434. 10.1038/s41420-022-01212-0 PMC9617873 36309489

[35] Doll S, Freitas FP, Shah R, Aldrovandi M, da Silva MC, Ingold I, et al. FSP1 is a glutathione-independent ferroptosis suppressor. Nature. 2019;575:693–8. 10.1038/s41586-019-1707-0 31634899

[36] Chu B, Kon N, Chen D, Li T, Liu T, Jiang L, et al. ALOX12 is required for p53-mediated tumour suppression through a distinct ferroptosis pathway. Nat Cell Biol. 2019;21:579–91. 10.1038/s41556-019-0305-6 PMC6624840 30962574

[37] Mao C, Liu X, Zhang Y, Lei G, Yan Y, Lee H, et al. DHODH-mediated ferroptosis defence is a targetable vulnerability in cancer. Nature. 2021;593:586–90. 10.1038/s41586-021-03539-7 PMC8895686 33981038

[38] Zou Y, Henry WS, Ricq EL, Graham ET, Phadnis VV, Maretich P, et al. Plasticity of ether lipids promotes ferroptosis susceptibility and evasion. Nature. 2020;585:603–8. 10.1038/s41586-020-2732-8 PMC8051864 32939090

[39] Liang D, Feng Y, Zandkarimi F, Wang H, Zhang Z, Kim J, et al. Ferroptosis surveillance independent of GPX4 and differentially regulated by sex hormones. Cell. 2023;186:2748–64.e22. 10.1016/j.cell.2023.05.003 PMC10330611 37267948

[40] Wenzel SE, Tyurina YY, Zhao J, St Croix CM, Dar HH, Mao G, et al. PEBP1 wardens ferroptosis by enabling lipoxygenase generation of lipid death signals. Cell. 2017;171:628–41.e26. 10.1016/j.cell.2017.09.044 PMC5683852 29053969

[41] Ma S, Sun R, Jiang B, Gao J, Deng W, Liu P, et al. L2hgdh deficiency accumulates l-2-hydroxyglutarate with progressive leukoencephalopathy and neurodegeneration. Mol Cell Biol. 2017;37:e00492-16. 10.1128/MCB.00492-16 PMC5376639 28137912

[42] Tabata S, Kojima Y, Sakamoto T, Igarashi K, Umetsu K, Ishikawa T, et al. L-2hydroxyglutaric acid rewires amino acid metabolism in colorectal cancer via the mTOR-ATF4 axis. Oncogene. 2023;42:1294–307. 10.1038/s41388-023-02632-7 PMC10101855 36879117

[43] Kundu A, Brinkley GJ, Nam H, Karki S, Kirkman R, Pandit M, et al. l-2-Hydroxyglutarate remodeling of the epigenome and epitranscriptome creates a metabolic vulnerability in kidney cancer models. J Clin Invest. 2024;134:e171294. 10.1172/JCI171294 PMC11213505 38743486

[44] Shelar S, Shim EH, Brinkley GJ, Kundu A, Carobbio F, Poston T, et al. Biochemical and epigenetic insights into L-2-hydroxyglutarate, a potential therapeutic target in renal cancer. Clin Cancer Res. 2018;24:6433–46. 10.1158/1078-0432.CCR-18-1727 PMC6295227 30108105

[45] Gu W, Wang X, Solmonson A, Cai L, Xiao Y, Tasdogan A, et al. L-2-hydroxyglutarate impairs neuronal differentiation through epigenetic activation of *MYC* expression. bioRxiv. 2025. 2025.04.16.649033. 10.1172/JCI197010 PMC13132370 41842973

[46] Qiu Z, Lin AP, Jiang S, Elkashef SM, Myers J, Srikantan S, et al. MYC Regulation of D2HGDH and L2HGDH influences the epigenome and epitranscriptome. Cell Chem Biol. 2020;27:538–50.e7. 10.1016/j.chembiol.2020.02.002 PMC7714266 32101699

[47] Wang TX, Liang JY, Zhang C, Xiong Y, Guan KL, Yuan HX. The oncometabolite 2-hydroxyglutarate produced by mutant IDH1 sensitizes cells to ferroptosis. Cell Death Dis. 2019;10:755. 10.1038/s41419-019-1984-4 PMC6779886 31591388

[48] Su R, Dong L, Li C, Nachtergaele S, Wunderlich M, Qing Y, et al. R-2HG exhibits anti-tumor activity by targeting FTO/m(6)A/MYC/CEBPA signaling. Cell. 2018;172:90–105.e23. 10.1016/j.cell.2017.11.031 PMC5766423 29249359

[49] Li R, Yan X, Xiao C, Wang T, Li X, Hu Z, et al. FTO deficiency in older livers exacerbates ferroptosis during ischaemia/reperfusion injury by upregulating ACSL4 and TFRC. Nat Commun. 2024;15:4760. 10.1038/s41467-024-49202-3 PMC11150474 38834654

[50] Qiao Y, Su M, Zhao H, Liu H, Wang C, Dai X, et al. Targeting FTO induces colorectal cancer ferroptotic cell death by decreasing SLC7A11/GPX4 expression. J Exp Clin Cancer Res. 2024;43:108. 10.1186/s13046-024-03032-9 PMC11005233 38600610

[51] Andrade J, Shi C, Costa ASH, Choi J, Kim J, Doddaballapur A, et al. Control of endothelial quiescence by FOXO-regulated metabolites. Nat Cell Biol. 2021;23:413–23. 10.1038/s41556-021-00637-6 PMC8032556 33795871

[52] Hao J, Huang Z, Zhang S, Song K, Wang J, Gao C, et al. Deciphering the multifaceted roles and clinical implications of 2-hydroxyglutarate in cancer. Pharm Res. 2024;209:107437. 10.1016/j.phrs.2024.107437 39349213

[53] Jezek P. 2-Hydroxyglutarate in cancer cells. Antioxid Redox Signal. 2020;33:903–26. 10.1089/ars.2019.7902 PMC7533892 31847543

[54] Yang Y, Li X, Liu F, Ma M, Yang Y, Ruan C, et al. Immunometabolite L-2-HG promotes epigenetic modification of exhausted T cells and improves antitumor immunity. JCI Insight. 2025;10:e174600. 10.1172/jci.insight.174600 PMC11981629 40043713

