# Supplementary material for: L-2-Hydroxyglutarate sensitizes ferroptosis through ATF3/CHAC1-mediated glutathione degradation in hepatocellular carcinoma
Source: Cell Death Dis. 2026 Jul 8;17(1):850. doi: 10.1038/s41419-026-09052-x (PMC13631258; doi:10.1038/s41419-026-09052-x)

Fig 1C

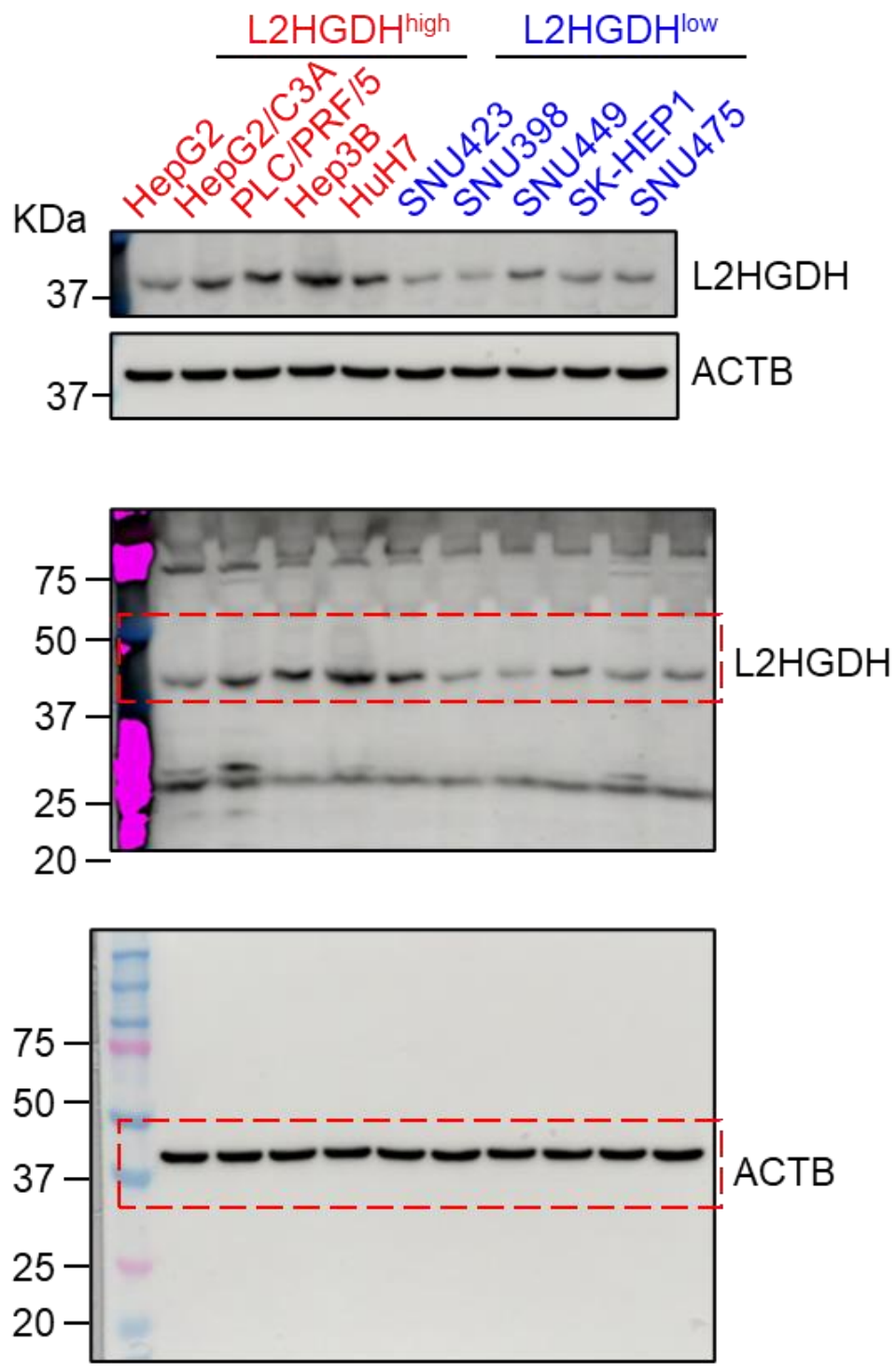
L2HGDHhigh
L2HGDHlow
HepG2
HepG2/C3A
PLC/PRF/5
Hep3B
HuH7
SNU423
SNU398
SNU449
SK-HEP1
SNU475
KDa
37
L2HGDH
37
ACTB
75
50
37
25
20
L2HGDH
75
50
37
25
20
ACTB

Fig 1K

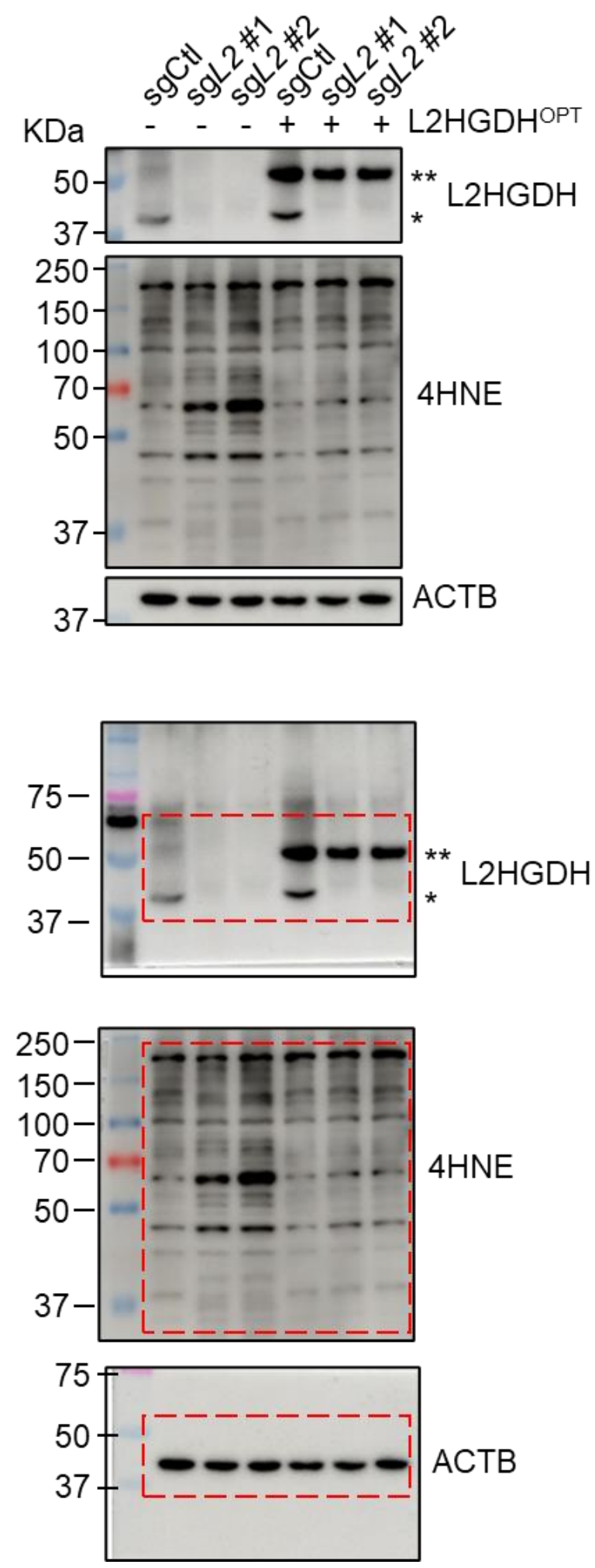
sgCtl
sgL2 #1
sgL2 #2
sgCtl
sgL2 #1
sgL2 #2
KDa
- - - + + + L2HGDHOPT
50
37
** L2HGDH
*
250
150
100
70
50
37
4HNE
37
ACTB
75
50
37
** L2HGDH
*
250
150
100
70
50
37
4HNE
75
50
37
ACTB

Supplemental Fig 1B

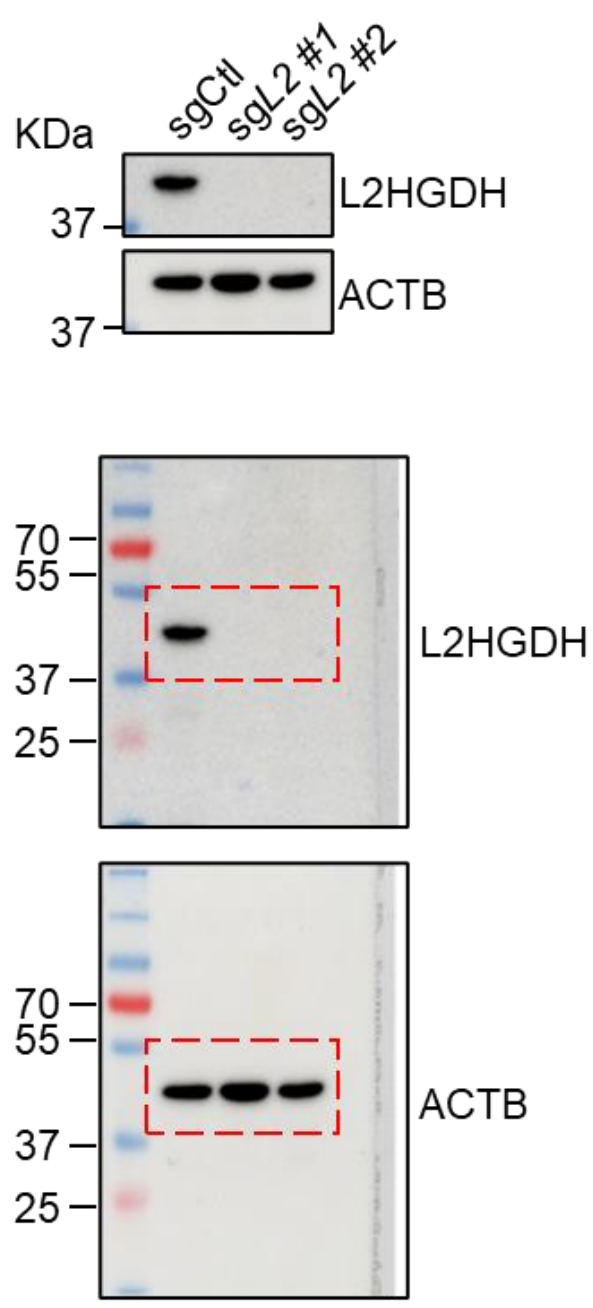

Supplemental Fig 1C

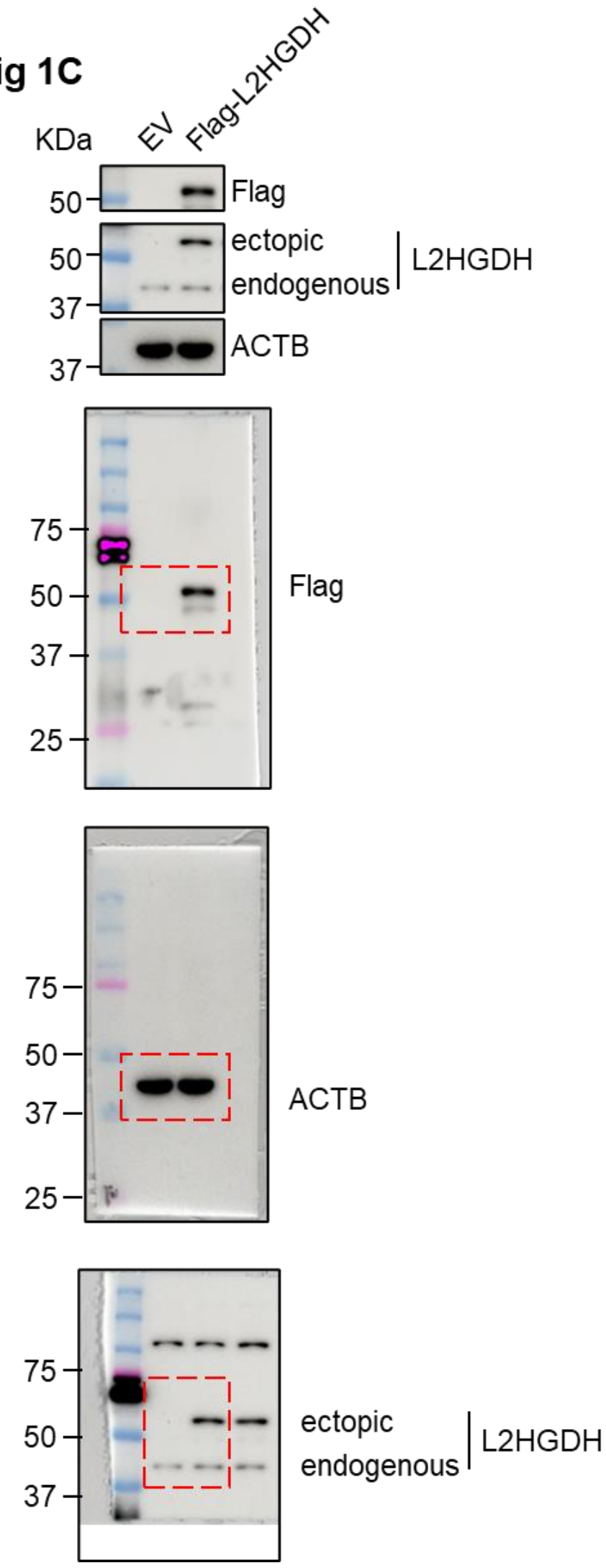

Fig 2N

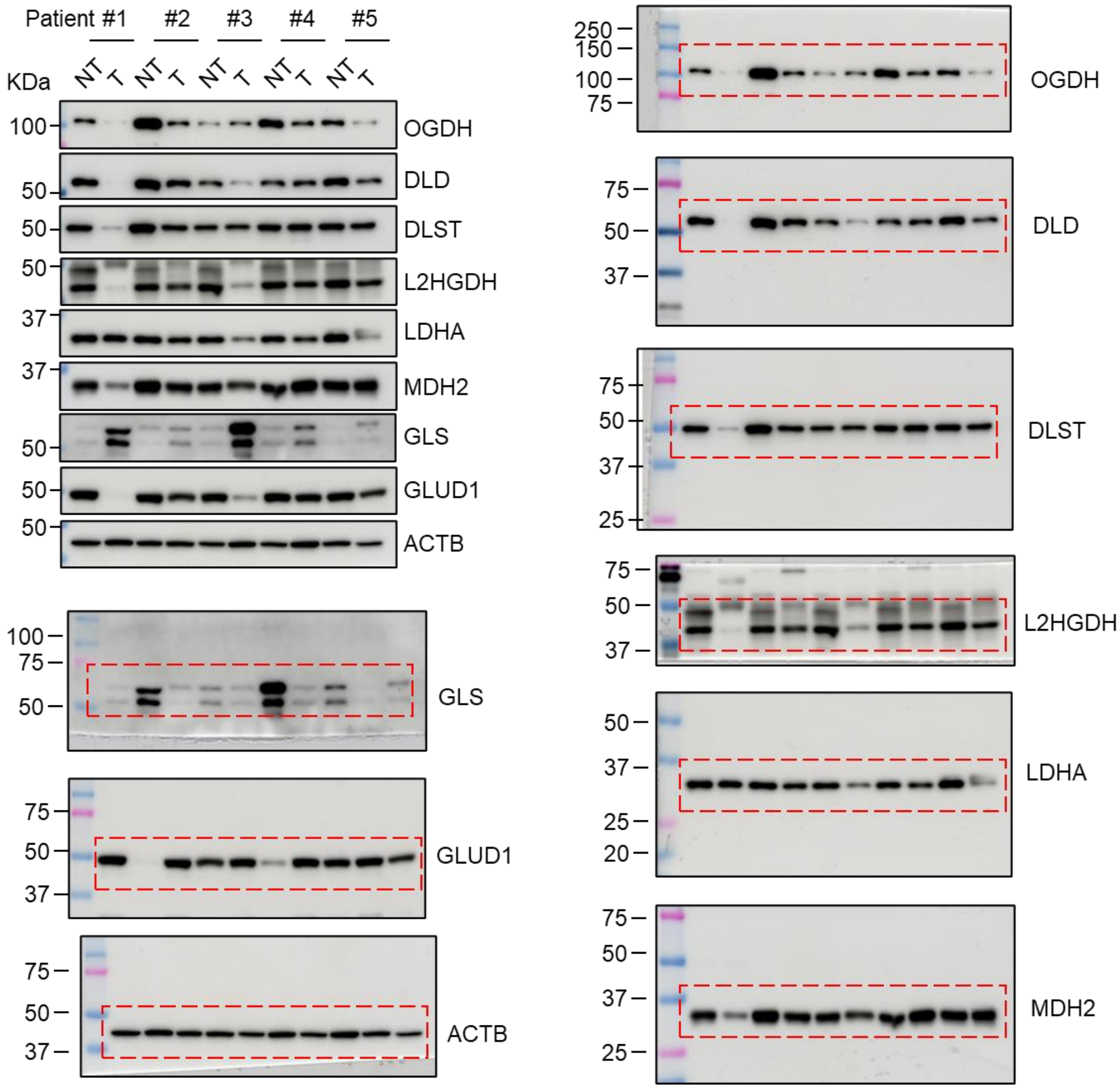

Patient #1
#2
#3
#4
#5
KDa
NT
T
100
50
37
OGDH
DLD
DLST
L2HGDH
LDHA
MDH2
GLS
GLUD1
ACTB
250
150
75
25
20

Supplemental Fig 2D

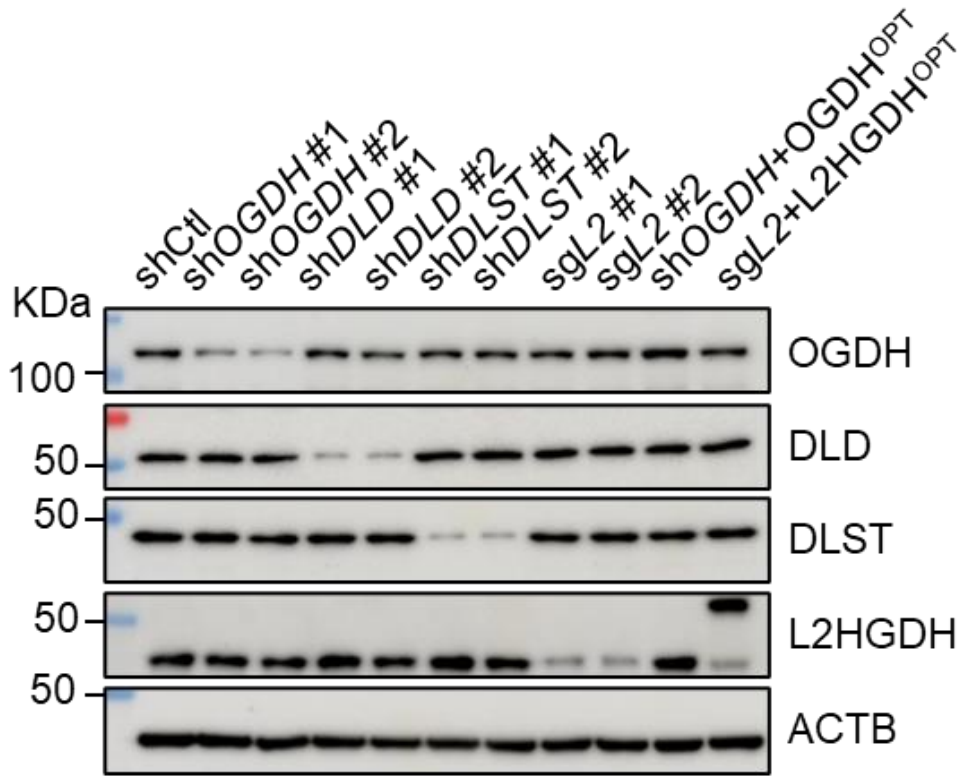

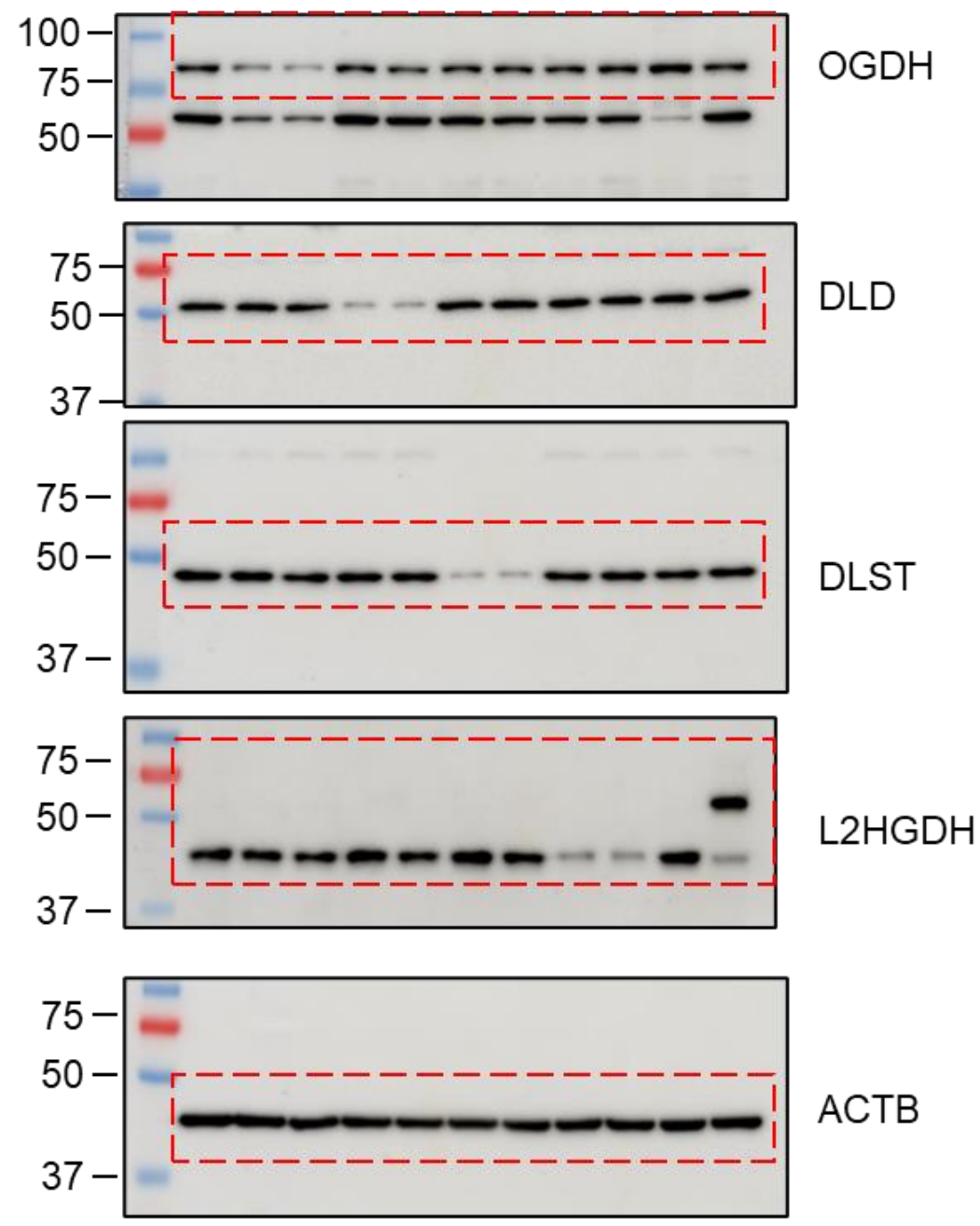

Fig 3D

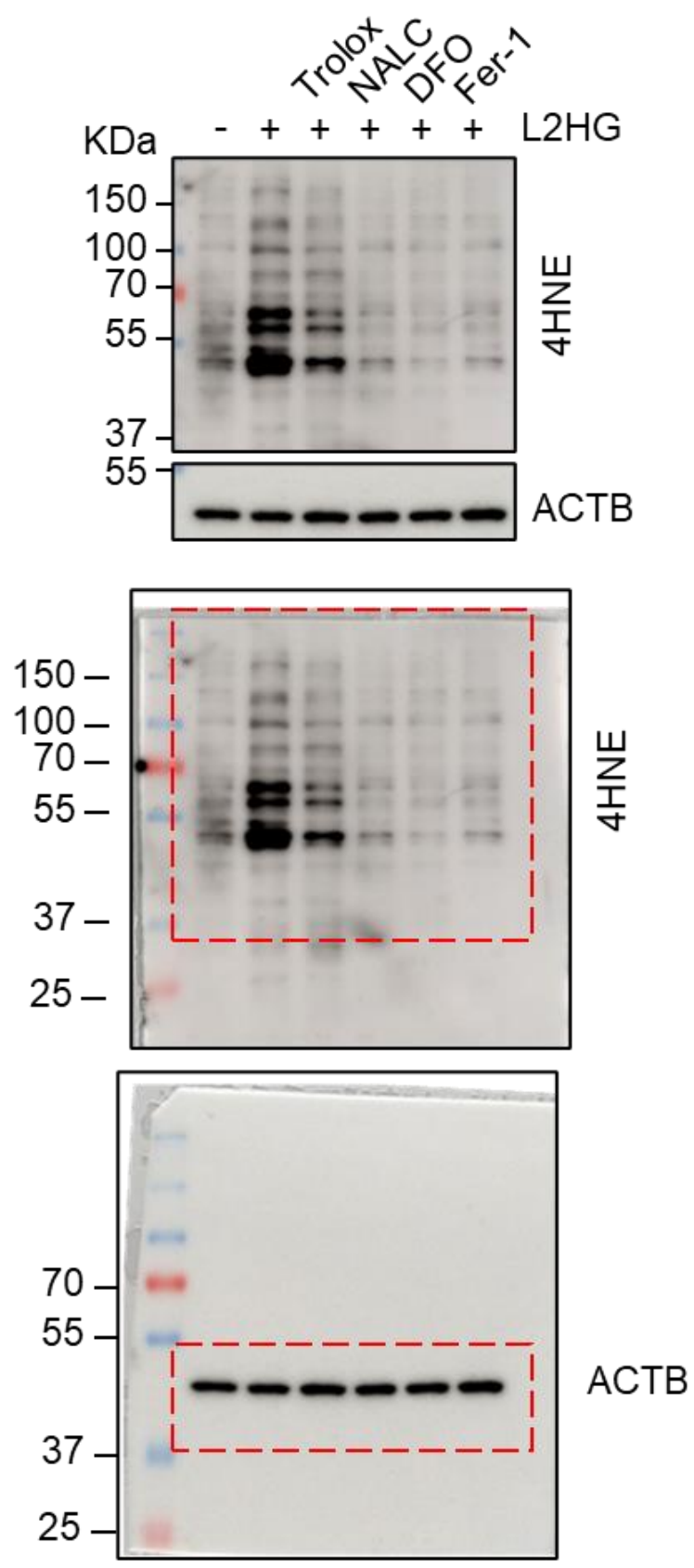
Trolox
NALC
DFO
Fer-1
KDa
- + + + + +
L2HG
150
100
70
55
37
4HNE
55
ACTB
150
100
70
55
37
25
4HNE
70
55
37
25
ACTB

Supplemental Fig 4B

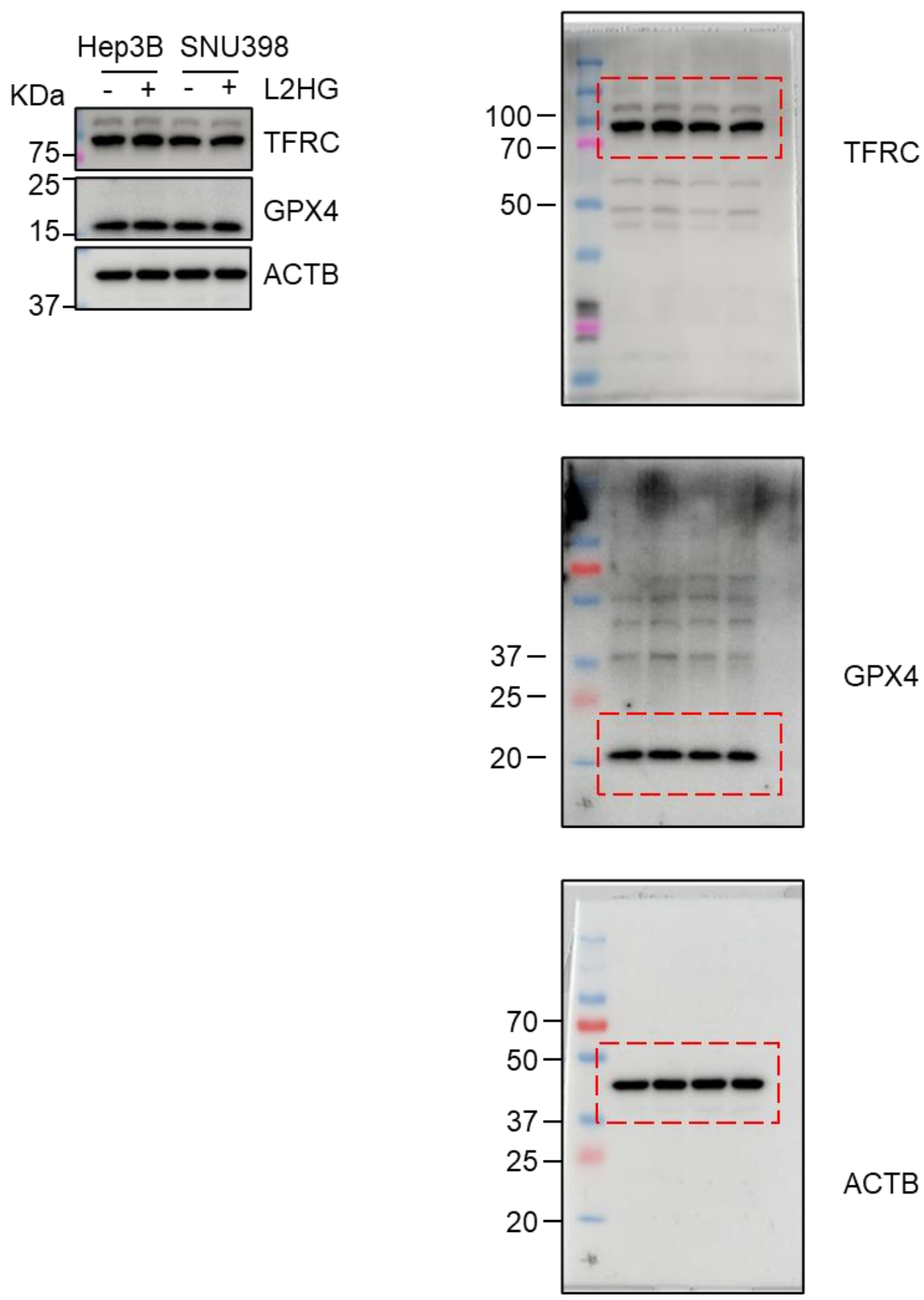

Fig 5D

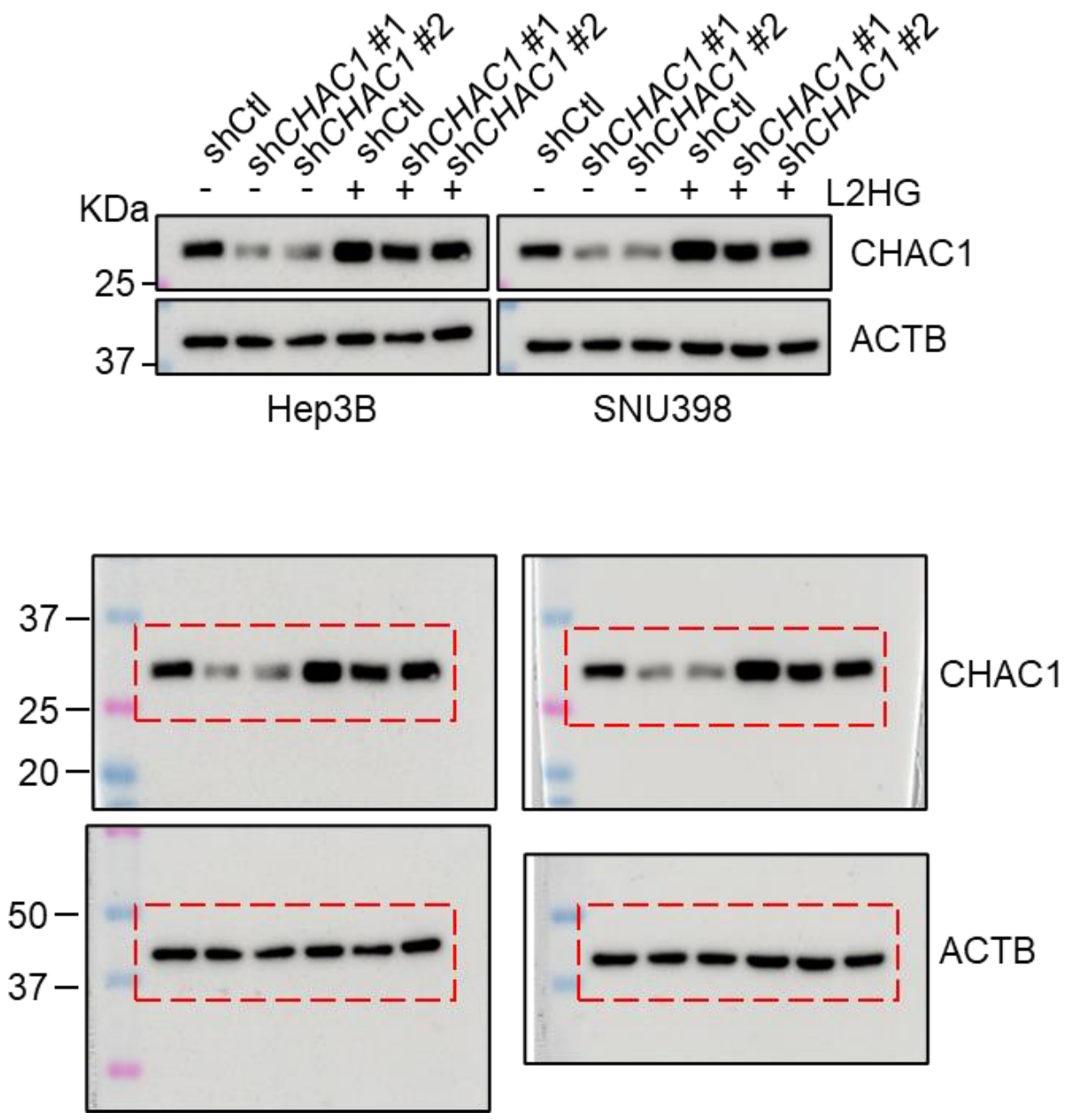

shCtl
shCHAC1 #1
shCHAC1 #2
shCtl
shCHAC1 #1
shCHAC1 #2
shCtl
shCHAC1 #1
shCHAC1 #2
shCtl
shCHAC1 #1
shCHAC1 #2
KDa
- - - + + +
- - - + + +
L2HG
CHAC1
25
ACTB
37
Hep3B
SNU398
37
25
20
CHAC1
50
37
ACTB

Fig 5F

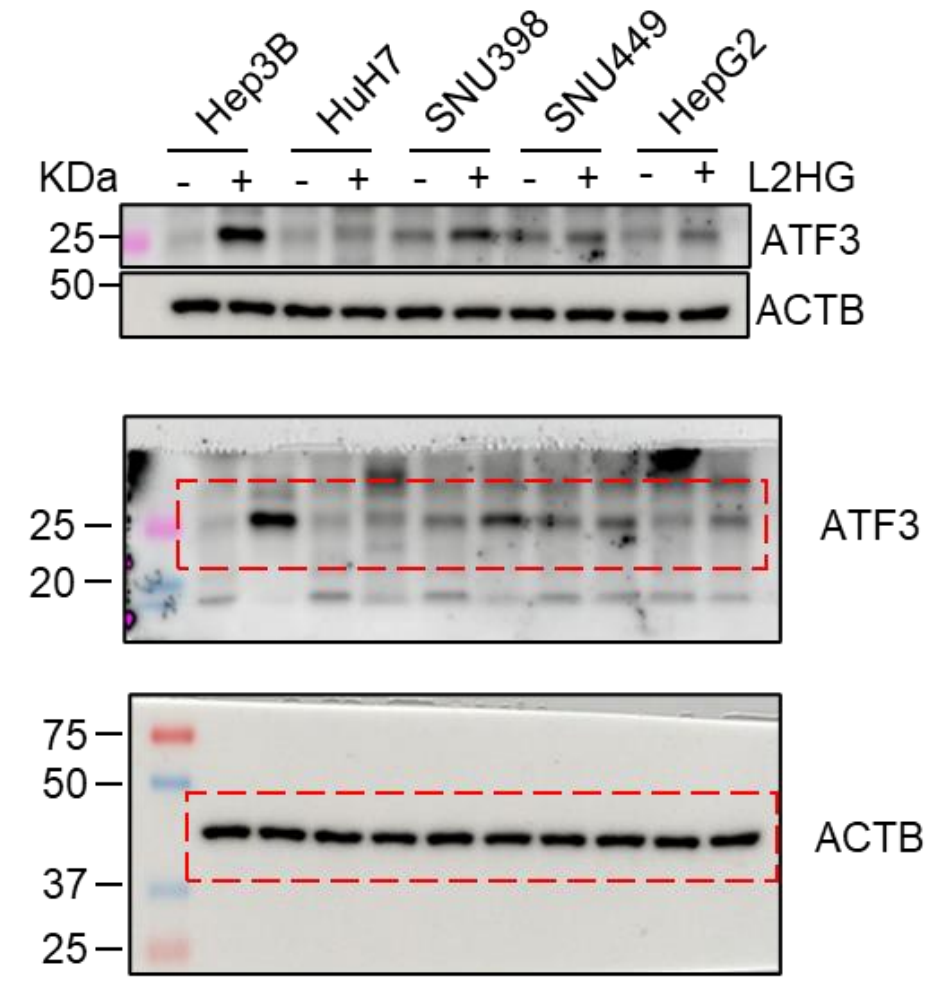

Hep3B
HuH7
SNU398
SNU449
HepG2
KDa
- + - + - + - + - +
L2HG
25
ATF3
50
ACTB
25
20
ATF3
75
50
37
25
ACTB

Supplemental Fig 5B

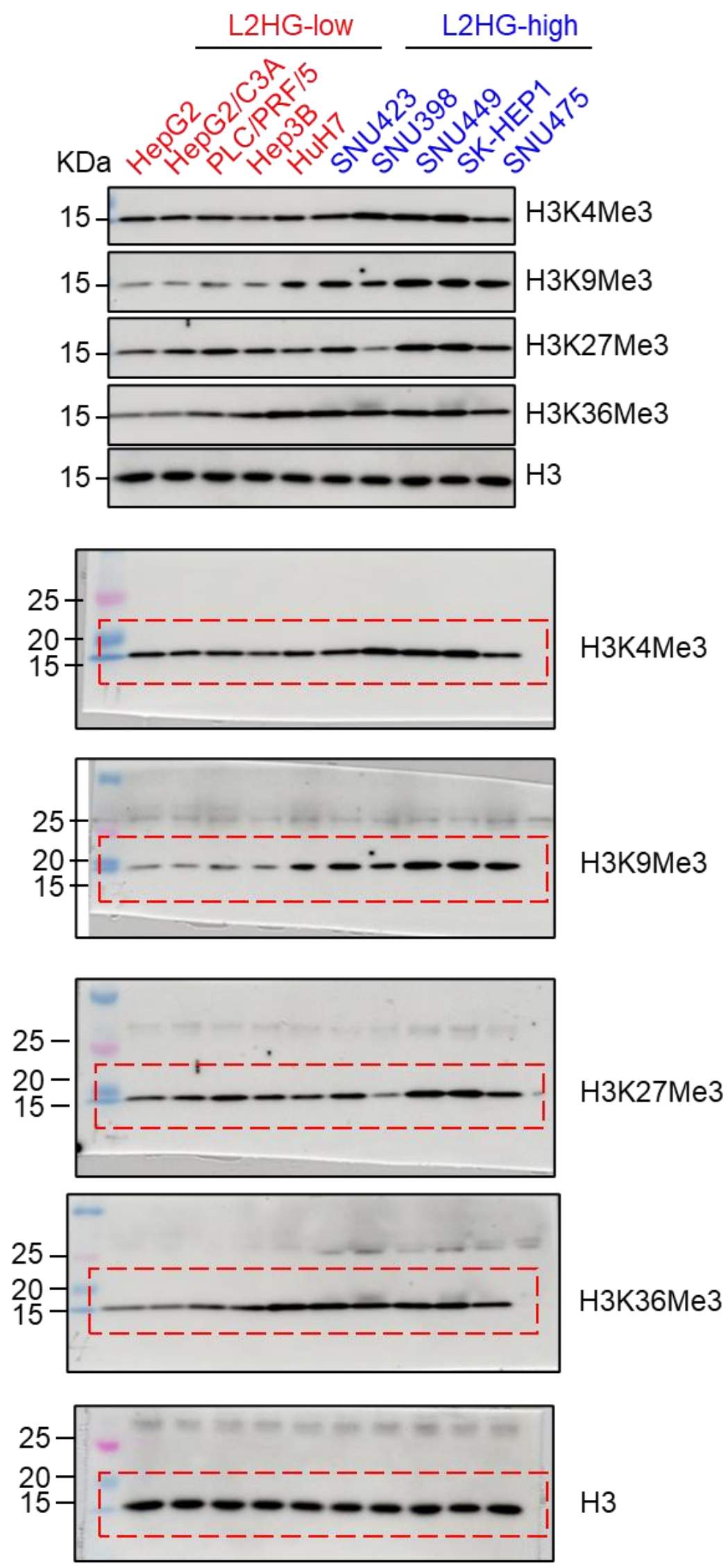

Supplemental Fig 5D

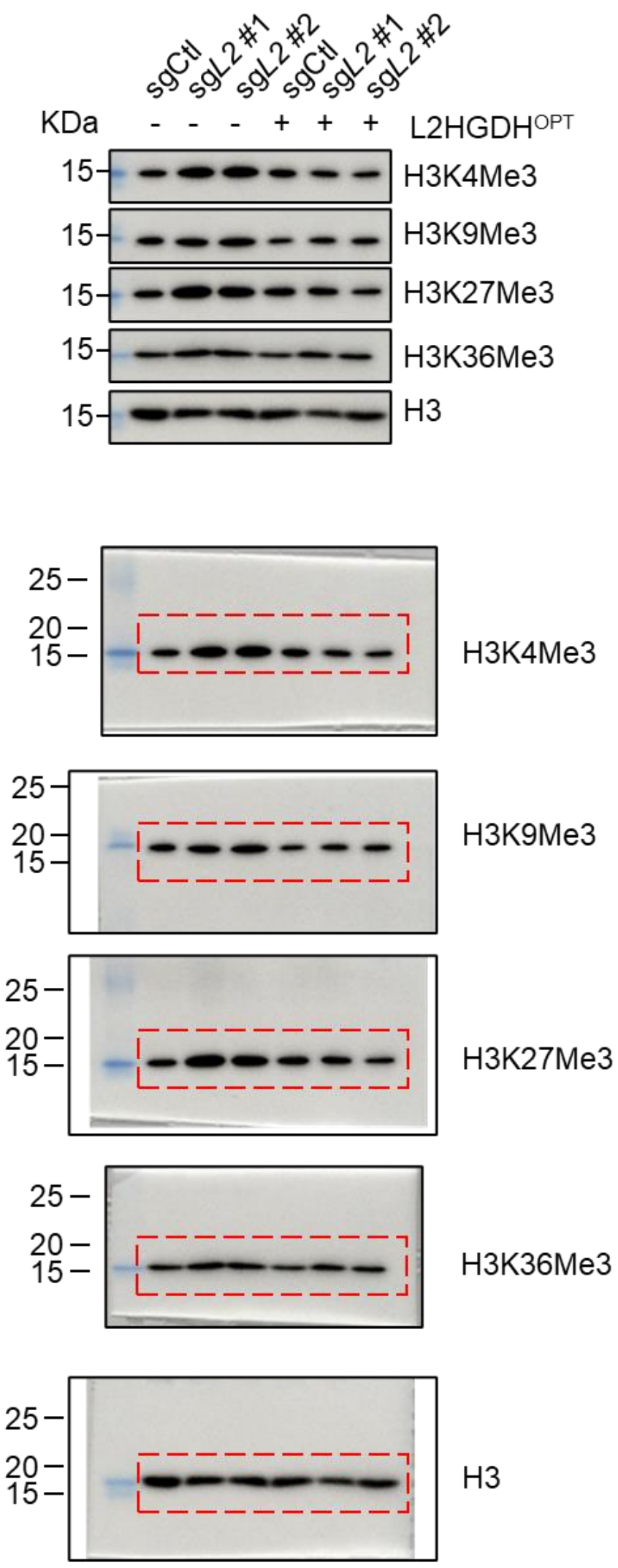

Supplemental Fig 5F

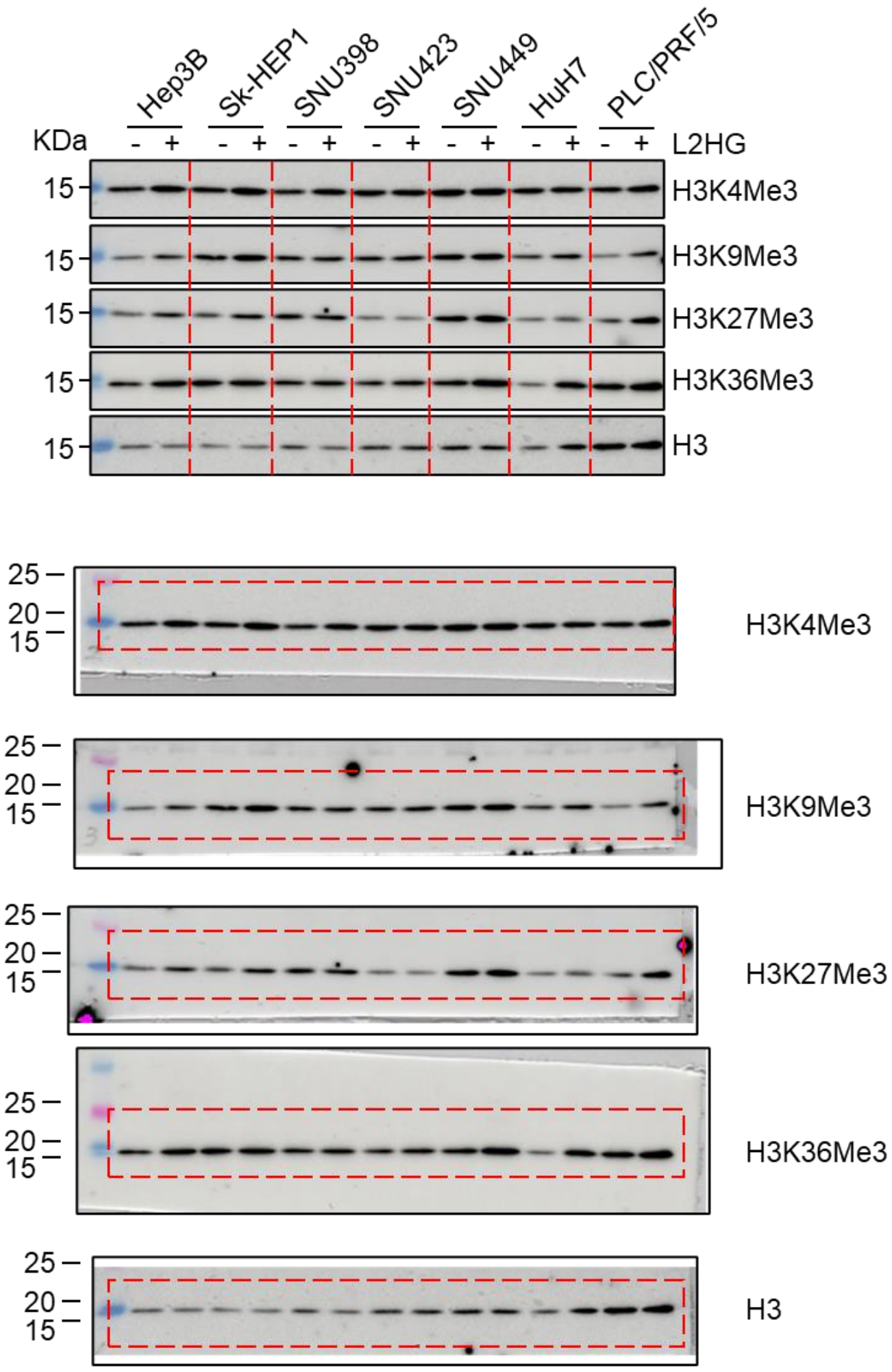

Supplemental Fig 5H

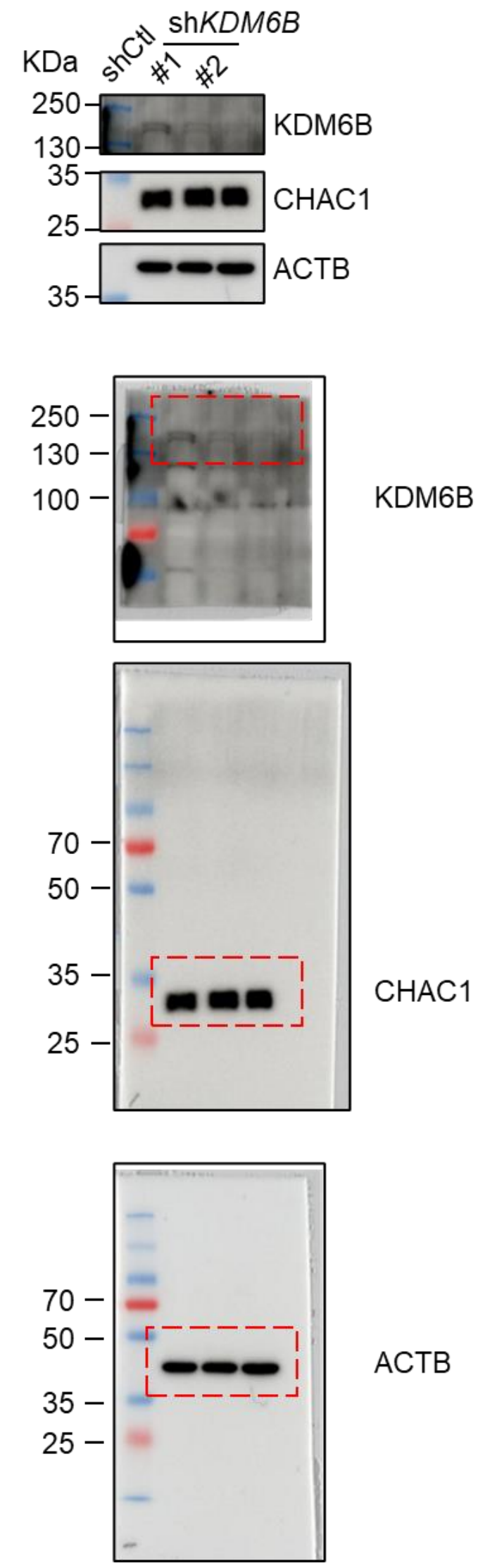

Supplemental Fig 6C

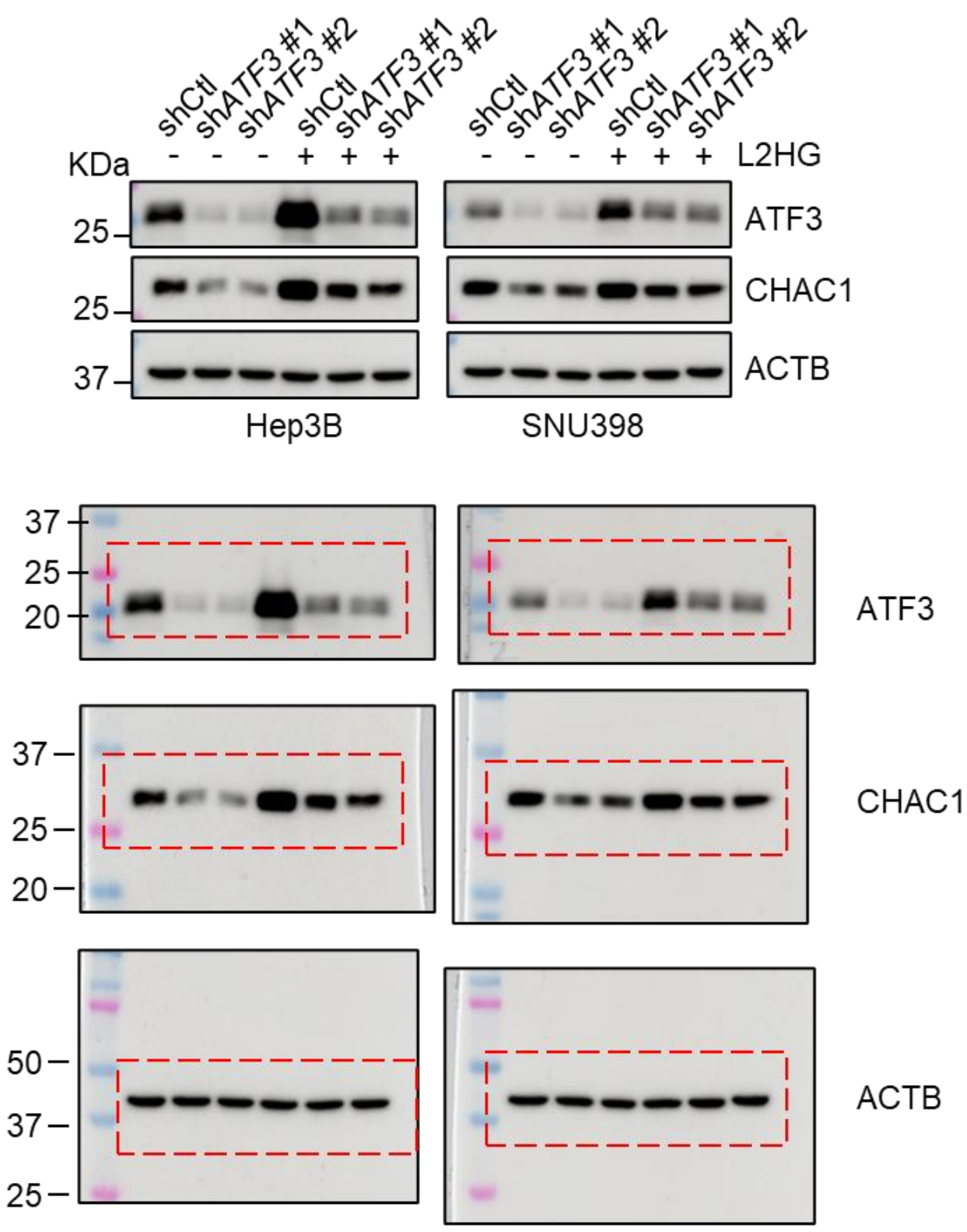

Fig 6J

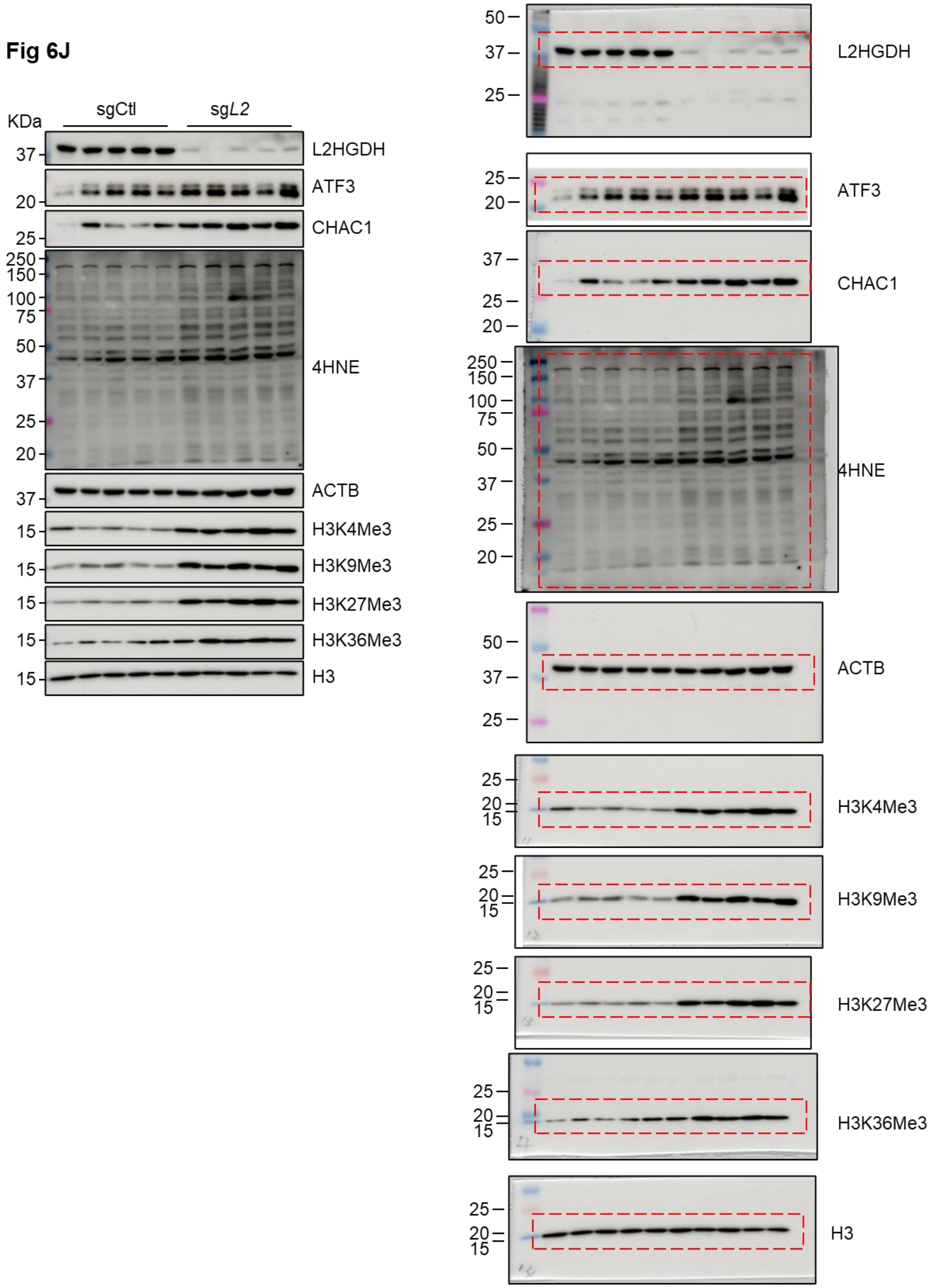

KDa
sgCtl
sgL2
L2HGDH
ATF3
CHAC1
4HNE
ACTB
H3K4Me3
H3K9Me3
H3K27Me3
H3K36Me3
H3
37
20
25
250
150
100
75
50
37
25
20
37
15
15
15
15
15

Flow cytometry gating strategy

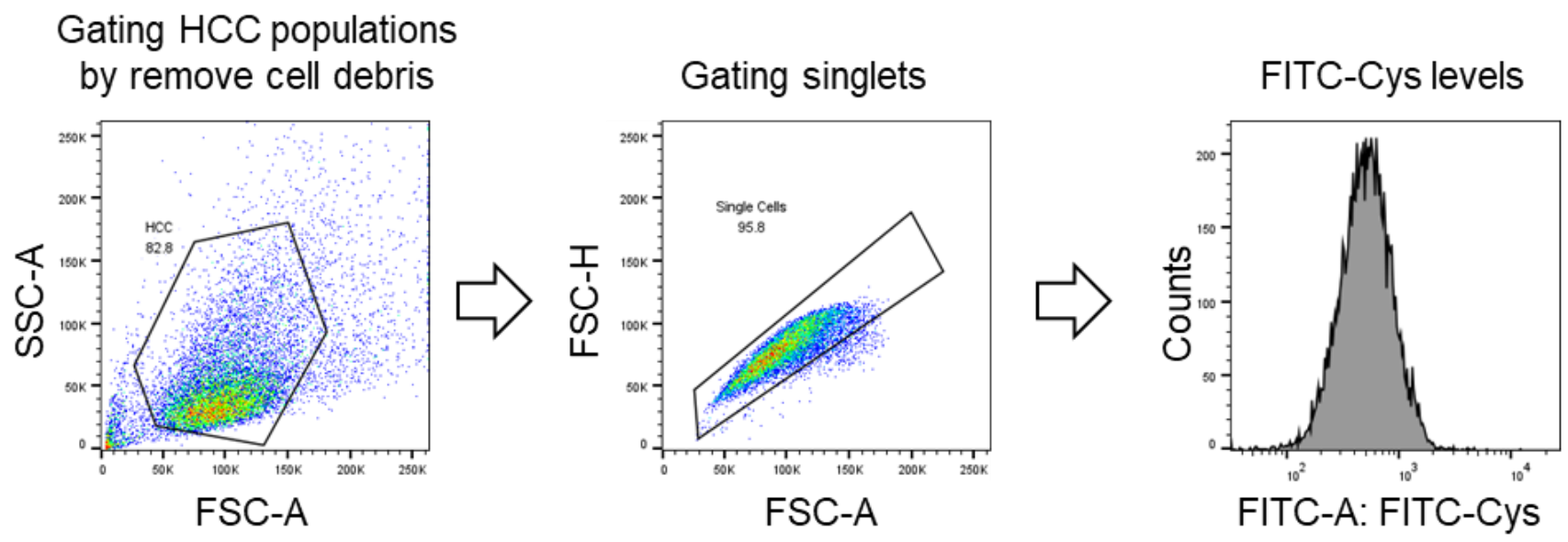

Supplement: Supplementary file 1 [file 41419_2026_9052_MOESM1_ESM.pdf]
