# Supplementary material for: L-2-Hydroxyglutarate sensitizes ferroptosis through ATF3/CHAC1-mediated glutathione degradation in hepatocellular carcinoma
Source: Cell Death Dis. 2026 Jul 8;17(1):850. doi: 10.1038/s41419-026-09052-x (PMC13631258; doi:10.1038/s41419-026-09052-x)
Supplement: Supplementary file 2 [file 41419_2026_9052_MOESM2_ESM.pdf]

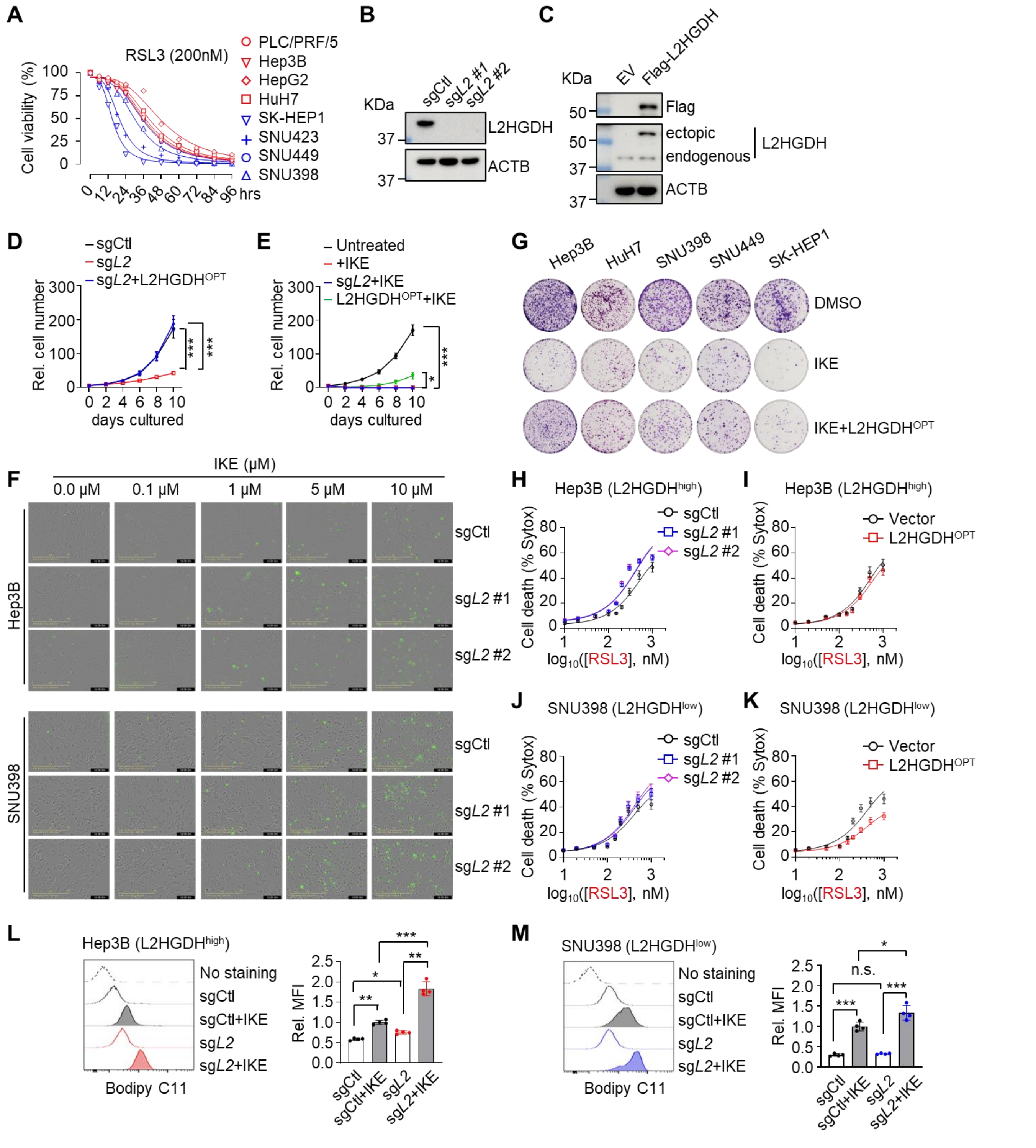

Supplementary Figure 1. L2HGDH antagonizes ferroptosis. **A**) Relative viability of L2HGDH^{high} and L2HGDH^{low} HCC cell lines following treatment with 200 nM RSL3 for the indicated times. **B**-**C**) Immunoblotting analysis of L2HGDH expression after sg*L2HGDH* (sg*L2*) knockout (**B**) or ectopic L2HGDH^{OPT} expression (**C**) in Hep3B cells. L2HGDH^{OPT} migrates ~7 kDa larger than endogenous L2HGDH. **D**-**E**) Relative cellular proliferation of Hep3B cells following sg*L2HGDH*, ectopic L2HGDH^{OPT} expression (**D**) or under ferroptosis induction with IKE (5 µM) (**E**). **F**) Representative microphotographs of Hep3B and SNU398 cells for cell death stained with Sytox green under sg*L2HGDH* (sg*L2*) and different concentrations of IKE treatment for 24 hours. **G**) Colony formation assays of HCC cells treated with IKE or L2HGDH^{OPT} expression. **H**-**K**) Incucyte Sytox green staining determined the cell death induced by different concentrations of RSL3 under *L2HGDH*

silencing (sg*L2HGDH*, sg*L2*) or ectopic L2HGDH^{OPT} expression in L2HGDH^{high} Hep3B and L2HGDH^{low} SNU398 cells. Two independent constructs sg*L2* were tested. **L**-**M**) Relative Bodipy-C11 fluorescence intensities in L2HGDH^{high} (Hep3B) and L2HGDH^{low} (SNU398) cell lines under IKE and sg*L2HGDH* silencing. The data are presented as the means of three different biological experiments. *$p < 0.05$, by unpaired Student's *t* test.

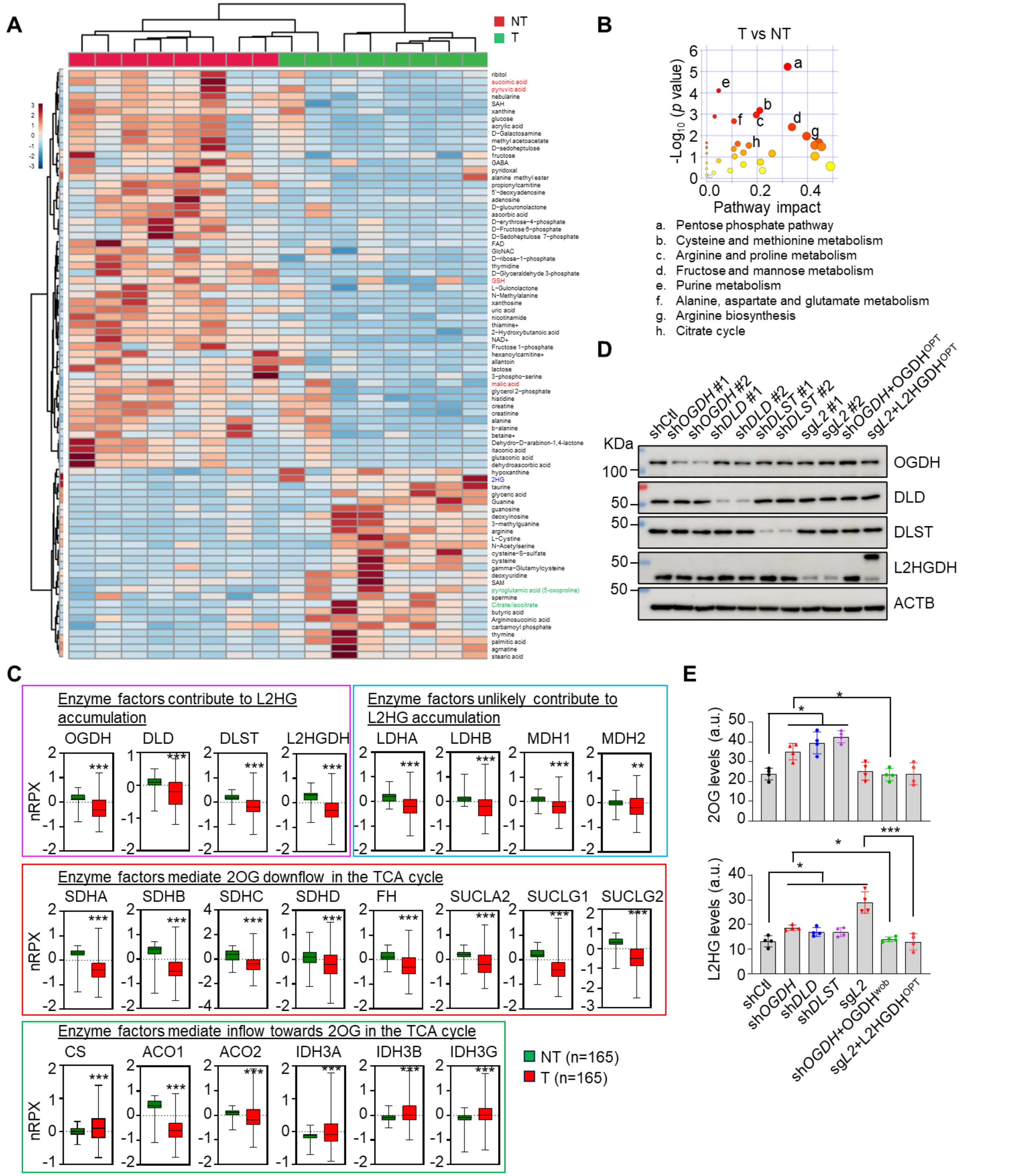

Supplementary Fig 2. HCC exhibits a metabolic regulatory network favoring L2HG accumulation. A-B) Heatmap (A) and pathway enrichment analysis (B) of the top 80 metabolites with significantly altered abundance between human HCC tumors (T) and adjacent non-tumor tissues (NT), analyzed using MetaboAnalyst 6.0 (n = 8 per group; $p < 0.05$). **C**) Normalized relative protein expression (nRPX) of enzymes involved in L2HG production and TCA cycle metabolism in patients' liver tumors. Data were retrieved from Human Protein Atlas datasets (https://www.proteinatlas.org/) (n=165 per group). **D-E**) Immunoblotting analysis of OGDH, DLD, DLST, and L2HGDH following shRNA/sgRNA and ectopic overexpression (OGDH^{OPT}

and L2HGDH^{OPT}) in Hep3B cells, with corresponding intracellular 2OG and L2HG levels quantified in (**E**) measured. The data is presented as mean ± SD from different biological experiments (n=3-8).

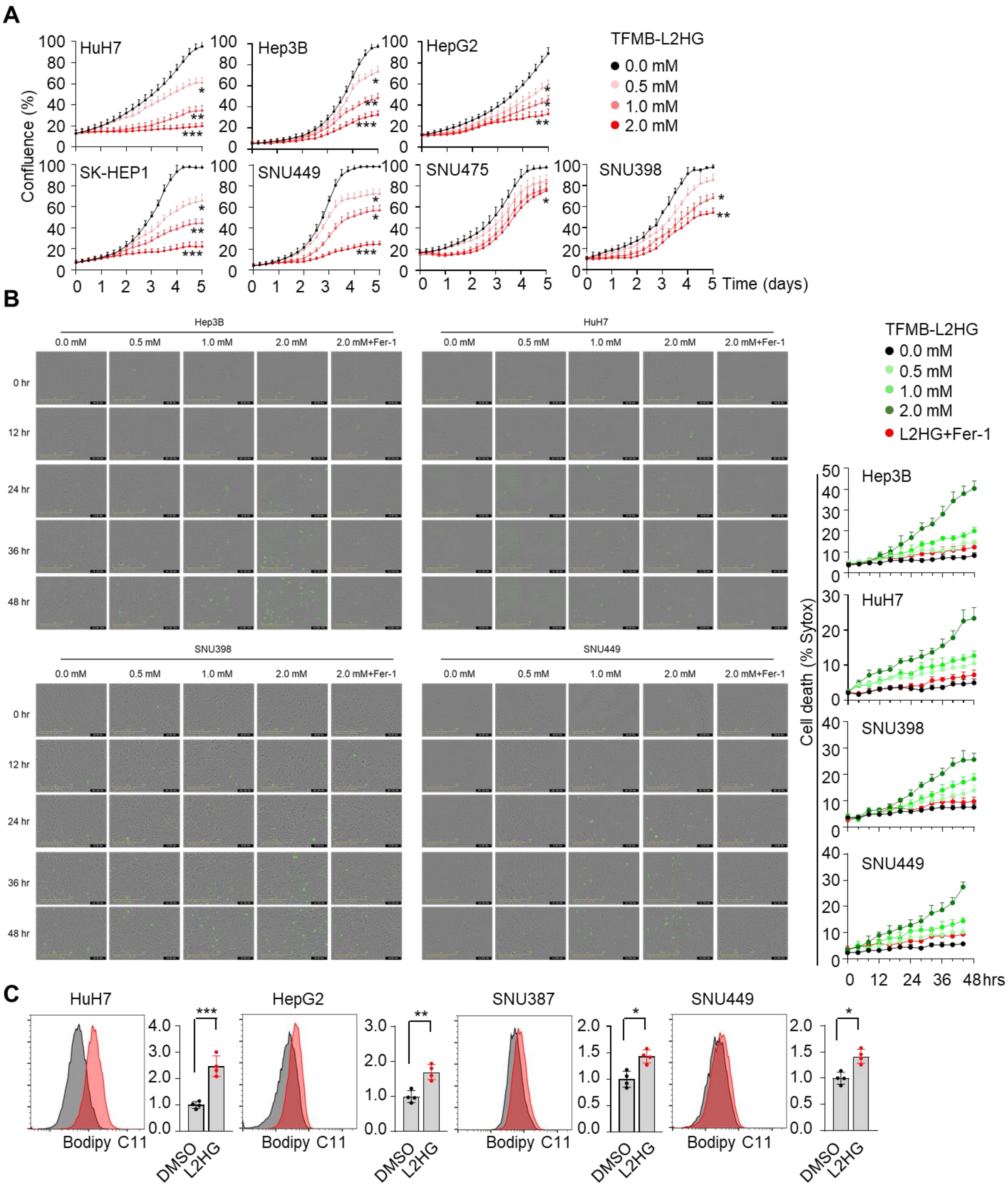

Supplementary Fig 3. L2HG induces ferroptosis in HCC cells. **A**) Incucyte analysis the proliferation of HCC cells following treatment with increasing concentrations of TFMB-L2HG (0-2 mM) over 5 days of culture. **B**) Representative microphotographs of L2HGDH^{high} (Hep3B and HuH7) and L2HGDH^{low} (SNU398 and SNU449) cells for cell death stained with Sytox green under increasing concentrations of TFMB-L2HG treatment (0-2 mM) along with ferroptosis inhibitor Fer-1 (1 μM) during 48 hours of culture. **C**) Flow cytometric analysis of lipid peroxidation using BODIPY-C11 staining in HCC cells treated with 2 mM TFMB-L2HG for 48 hours, with relative mean fluorescence intensity (MFI) quantified. The data is presented as mean ± SD from different biological experiments (n=3-4). *p < 0.05, **p < 0.01, ***p < 0.001.

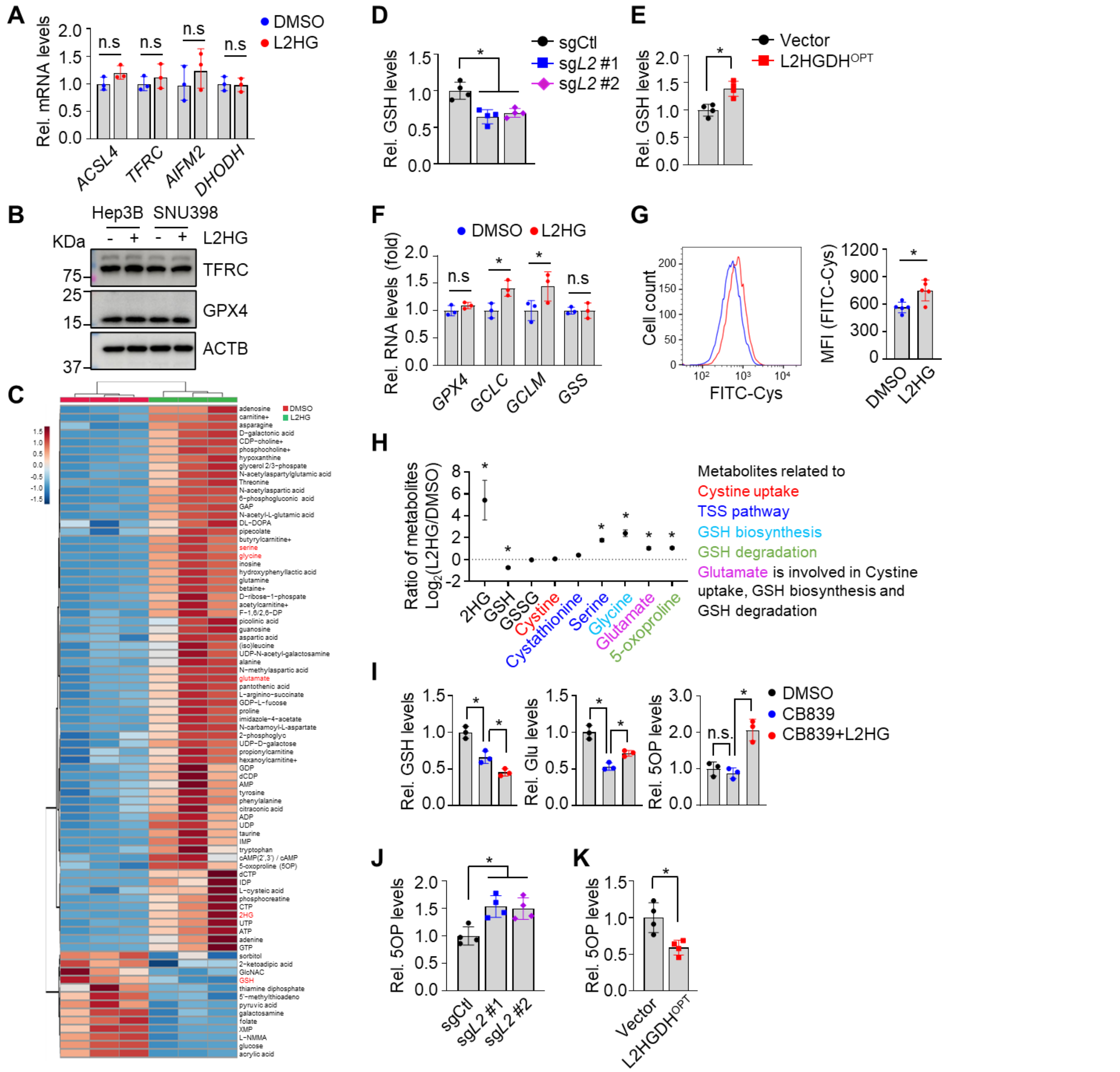

Supplementary Fig 4. L2HG disrupts GSH homeostasis. **A-B**) RT-PCR and western blot analyses determined the mRNA and protein expression of selected ferroptosis regulators under the treatment of TFMB-L2HG in Hep3B and SNU398 cells. **C**) Heatmap analysis of the top 80 metabolites with significantly altered abundance in Hep3B cells following TFMB-L2HG treatment (2 mM) using MetaboAnalyst 6.0. **D-E**) Relative intracellular GSH levels following sgRNA-mediated L2HGDH silencing (**D**) or ectopic $L2HGDH^{OPT}$ expression (**E**). **F**) qRT-PCR analysis of mRNA levels of enzymes involved in GSH synthesis and utilization. **G**) Measurement of cystine uptake in Hep3B cells treated with TFMB-L2HG using BioTracker FITC-cystine, with mean fluorescence intensity quantified. **H**) relative changes in metabolites involved in GSH homeostasis expressed as log_2[fold change (L2HG/DMSO)]. **I**) Effects of glutaminase inhibition (CB839, 4 µM) and TFMB-L2HG (2 mM) on intracellular GSH, GSSG levels, and the GSH/GSSG ratio. **J-K**) Relative intracellular 5-oxoproline (5OP) levels following sg*L2HGDH* silencing (**J**) or ectopic $L2HGDH^{OPT}$ expression (**K**). The data is presented as mean ± SD of 3 different biological experiments. *$p < 0.05$.

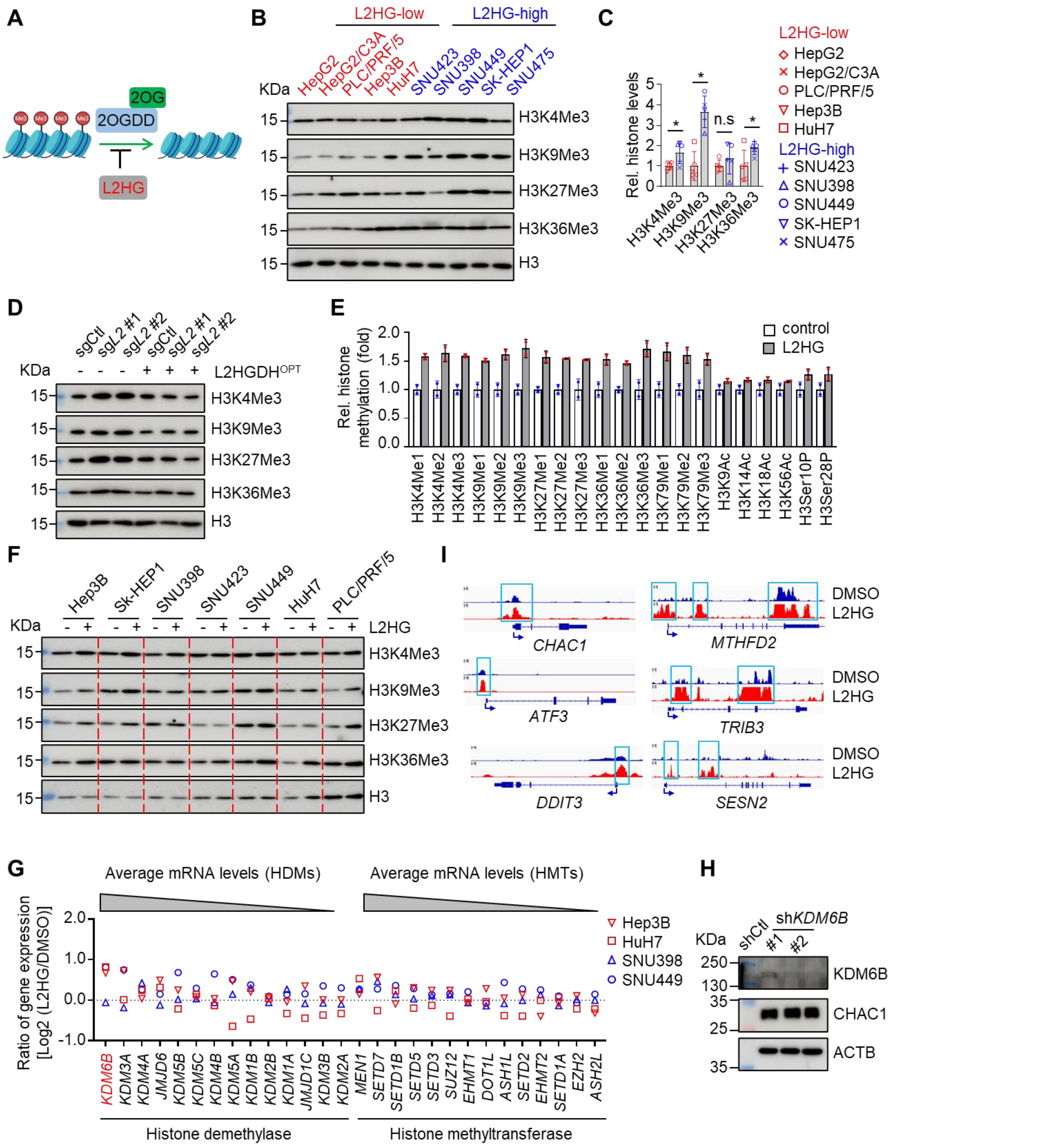

Supplementary Figure 5. L2HG induces histone hypermethylation. **A**) Schematic illustrating histone demethylation mediated by 2-oxoglutarate-dependent dioxygenases (2OGDDs) using 2OG as a cofactor, and competitive inhibition of this process by L2HG. **B-C**) Immunoblotting analysis and quantification of histone methylation codes in L2HG-low (L2HGDH^{high}) and L2HG-high (L2HGDH^{low}) HCC cell lines. **D**) Effects of L2HGDH silencing (sh*L2HGDH*) or ectopic L2HGDH^{OPT} expression on histone methylation in Hep3B cells. **E**) Effects of TFMB-L2HG on histone H3 methylation in Hep3B cells measured using a histone modification multiplex assay. **F**) Immunoblot analysis of histone methylation marks in multiple HCC cell lines following TFMB-L2HG treatment. **G**) Relative mRNA expression levels of indicated histone demethylation and methyltransferase genes under TFMB-L2HG treatment for 48 hours in different HCC cell lines, compared with DMSO control, shown as $\log_2$[fold change (L2HG/DMSO)]. **H**) Protein western blot

analyses determined the expression of KDM6B and CHAC1 after silence (shKDM6B) in Hep3B cells. **I**) ATAC-seq analysis the chromatin accessibility in ChAC1, ATF3 and target loci. The schematic of genes is shown below the panels with the arrow indicating the transcription start site.

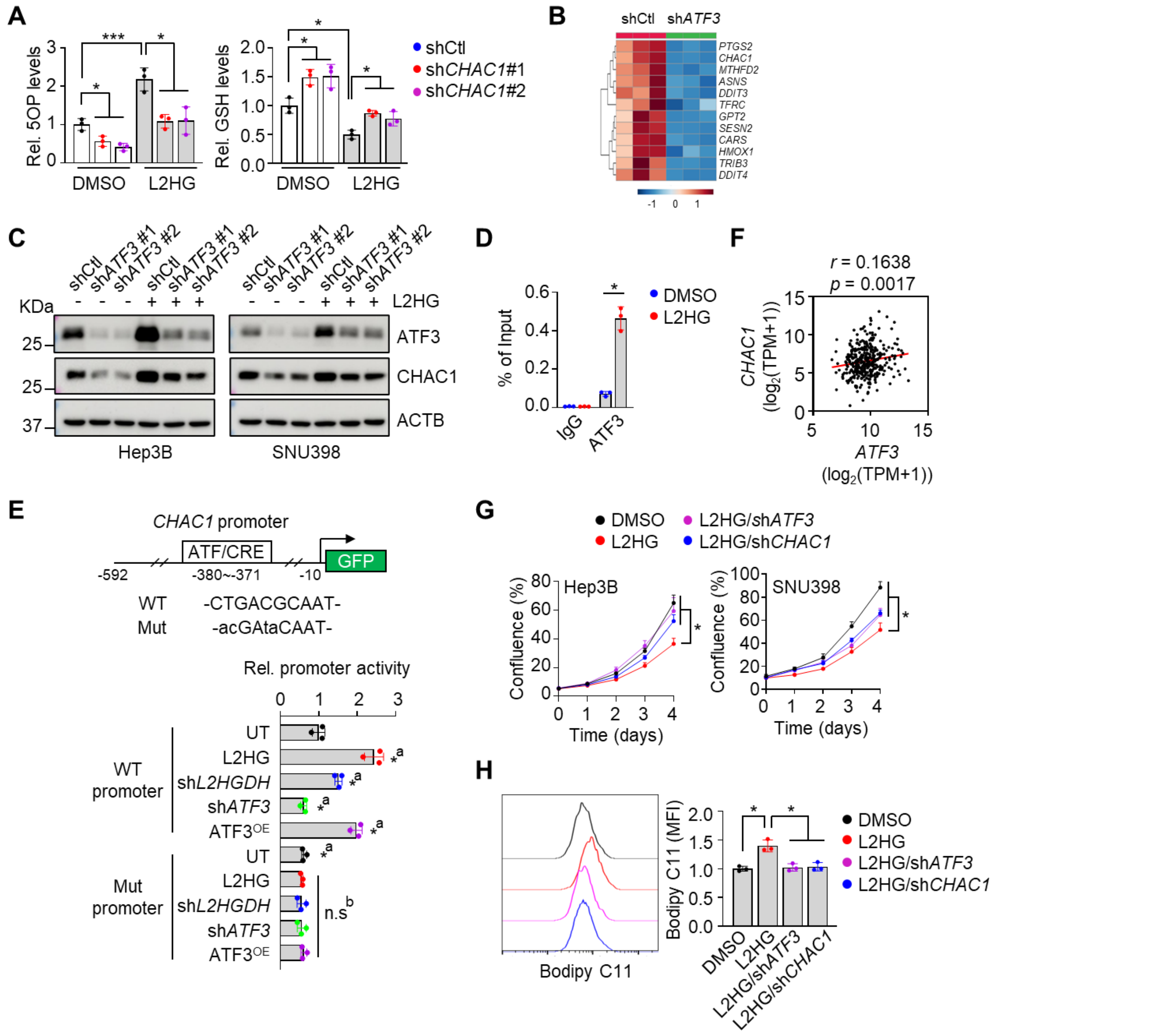

Supplementary Figure 6. The ATF3/CHAC1 axis regulates 5-oxoproline production. **A**) Effects of CHAC1 knockdown (sh*CHAC1*) on intracellular 5OP and GSH levels in SNU398 cells treated with TFMB-L2HG. **B**) Effects of ATF3 knockdown (sh*ATF3*) on ferroptosis-related gene expression. **C**) Immunoblot analysis of sh*ATF3* knockdown on ATF3 and CHAC1 protein expression in Hep3B and SNU398 cells under TFMB-L2HG. **D**) ChIP analysis of ATF3 binding to the *CHAC1* promoter in Hep3B cells treated with TFMB-L2HG. **E**) promoter activity assay for CHAC1 wild type and ATF/CRE motif mutant constructs in Hep3B cells under different conditions including L2HG treatment, silencing and ectopic overexpression of L2HGDH and ATF3 (sh*L2HGDH*, sh*ATF3*, ATF3^{OE}). a and b, statistical analysis was conducted against untreated (UT) control of WT promoter (a) or mutant promoter (b). **F**) Correlation analysis of CHAC1 and ATF3 mRNA expression in human liver tumor samples using data from the TCGA-LIHC dataset. **G**) Relative cellular proliferation of Hep3B and SNU398 cells treated with TFMB-L2HG in combination with sg*ATF3* and sg*CHAC1*. **H**) Lipid peroxidation assessed by BODIPY-C11 staining in SNU398 cells treated with TFMB-L2HG, sh*ATF3*, or sh*CHAC1*, with relative MFI quantified. The data is presented as mean ± SD of 3 different biological experiments. *$p < 0.05$.
